# A simple method for identifying parameter correlations in partially observed linear dynamic models

**DOI:** 10.1186/s12918-015-0234-3

**Published:** 2015-12-14

**Authors:** Pu Li, Quoc Dong Vu

**Affiliations:** Department of Simulation and Optimal Processes, Institute of Automation and Systems Engineering, Technische Universität Ilmenau, P. O. Box 100565, 98684 Ilmenau, Germany

**Keywords:** Linear model, Parameter estimation, Identifiability analysis, Parameter correlation, Remedy of non-identifiability, Experimental design

## Abstract

**Background:**

Parameter estimation represents one of the most significant challenges in systems biology. This is because biological models commonly contain a large number of parameters among which there may be functional interrelationships, thus leading to the problem of non-identifiability. Although identifiability analysis has been extensively studied by analytical as well as numerical approaches, systematic methods for remedying practically non-identifiable models have rarely been investigated.

**Results:**

We propose a simple method for identifying pairwise correlations and higher order interrelationships of parameters in partially observed linear dynamic models. This is made by derivation of the output sensitivity matrix and analysis of the linear dependencies of its columns. Consequently, analytical relations between the identifiability of the model parameters and the initial conditions as well as the input functions can be achieved. In the case of structural non-identifiability, identifiable combinations can be obtained by solving the resulting homogenous linear equations. In the case of practical non-identifiability, experiment conditions (i.e. initial condition and constant control signals) can be provided which are necessary for remedying the non-identifiability and unique parameter estimation. It is noted that the approach does not consider noisy data. In this way, the practical non-identifiability issue, which is popular for linear biological models, can be remedied. Several linear compartment models including an insulin receptor dynamics model are taken to illustrate the application of the proposed approach.

**Conclusions:**

Both structural and practical identifiability of partially observed linear dynamic models can be clarified by the proposed method. The result of this method provides important information for experimental design to remedy the practical non-identifiability if applicable. The derivation of the method is straightforward and thus the algorithm can be easily implemented into a software packet.

**Electronic supplementary material:**

The online version of this article (doi:10.1186/s12918-015-0234-3) contains supplementary material, which is available to authorized users.

## Background

Model-based analysis and synthesis become increasingly important in systems biology [1–4]. However, a significant obstacle to effective model-based studies comes from the fact that it is highly difficult to achieve the values of model parameters. A straightforward way to address this issue is to fit the model to the measured data by proper experimental design [5–7]. Nevertheless, such a fitting may usually fail, even for quite simple models, because some parameters in the model can be non-identifiable.

There are two major reasons for model non-identifiability. On the one hand, many biological models contain a large number of parameters to be estimated, among which some parameters may have functional interrelationships. This is largely because of the model structure when considering compound reaction networks [8–10]. More important is that biologists experiment with living cells and therefore the possibilities to stimulate the cells are limited, i.e. the control signals and the initial condition cannot be chosen at will. This limitation leads to challenges in parameter estimation [11–13], in particular, the model can be non-identifiable, which is the concern of this paper.

As a consequence, the effect of one parameter will compensate that of another. These parameters are correlated each other and therefore non-identifiable [14, 15]. On the other hand, because of limiting experimental facility, the measured data for parameter estimation are incomplete; in particular, in many cases only a part of the variables in the model can be measured. The input-output relations of a partially observed model may become highly complicated and lead to implicit parameter correlations, even if the model structure itself contains no functional relationships between the parameters [16, 17].

The existence of parameter correlations will lead to enormous difficulties in estimating the model parameters. As a consequence, the parameters cannot be correctly constrained, the landscape of the predicting errors is quite flat, and the sensitivities of the resulting parameters are sloppy [18–20]. Therefore, identifiability analysis presents a very important and necessary task before performing parameter estimation. Although identifiability analysis of biological models has been extensively investigated in the past, the issue is far from being satisfactorily addressed [21–23].

In general, non-identifiable parameters in a model can be classified into structurally and practically non-identifiable parameters. Structurally non-identifiable parameters are those which cannot be estimated based on any measurement data with any quality and quantity, since the parameter correlations in effect are independent of the experimental condition. In contrast, the interrelationships of practically non-identifiable parameters are either due to a partial observation or due to inaccurate data [16]. As a result, it is possible to uniquely estimate the parameters based on datasets generated in experimental design by properly determining the experimental condition and improving the quality of the measurement. In this way, the non-identifiability of the model can be remedied. The “practical identifiability” in this paper means the case in which a non-identifiable model becomes identifiable if the initial condition and control signals are properly selected and meanwhile if the measured datasets are noise-free. Thus this provides a *necessary* condition for a practically identifiable model, i.e. even if this condition is satisfied, the model can be non-identifiable because of noisy data.

Therefore, the aim of identifiability analysis should be not only to figure out the structural and/or practical identifiability of the model but also, most importantly, to find the dependences of the identifiability on experimental conditions, so that proper experiments can be designed to remedy the non-identifiability when indicated. In particular, the impact of control signals and the initial condition of the identifiability has rarely been emphasized in the previous studies.

Linear ordinary differential equations (ODEs) are widely used to describe biological systems [24]. Many studies on identifiability analysis of linear dynamic models have been made and, in particular, methods for determining the structural identifiability have been developed for partially observed models [24–29]. The Laplace transformation methods were used for identifiability analysis of linear models [30, 31], but these studies did not consider parameter correlations based on analyzing the output sensitivity matrix. Another aspect in this area is the detection of explicit identifiable combinations of non-identifiable parameters which can help for model reparameterization [32–35]. More recently, identifiability conditions of *fully* observed linear models from one single dataset were presented; these conditions are related to the initial conditions of the system [36].

Using differential algebra, *a priori* methods were proposed to analyze structural identifiability of linear and nonlinear biological models without any requirement of measurements [33–35, 37–40], based on which effective software tools have been established [35, 41, 42]. In [43] it was pointed out that problems can arise by using differential algebra methods in testing the identifiability of systems started at some special initial conditions. However, from the previous studies for structural identifiability there are no suitable measures developed for remedying existing non-identifiability of a model. As a consequence, the accessible software tools will fail to run when unexpected but meaningful initial conditions and/or controls are provided, even for very simple linear models [35].

The measured data are always associated with noisiness and limitation of the number of sampling points which also make model parameters non-identifiable [8, 16]. Therefore, *a posteriori* methods detect the identifiability of a model by numerically solving the fitting problem based on available data [16, 17, 44, 45]. Using the method of profile likelihood, it is possible to find pairwise functional relations of the non-identifiable parameters if the corresponding manifold is one-dimensional [16]. Since the initial concentrations can be regarded as parameters to be estimated, the relation of the non-identifiability to the initial condition can be numerically characterized. As a result, the non-identifiability due to correlations can be remedied by means of defining proper initial conditions.

In a recent study [15], we proposed a method that is able to analytically identify both pairwise parameter correlations and higher order interrelationships among parameters in nonlinear dynamic models. Correlations are interpreted as surfaces in the subspaces of correlated parameters. Explicit relations between the parameter correlations and the control signals can be derived by simply analyzing the sensitivity matrix of the right-hand side of the model equations. Based on the correlation information obtained in this way both structural and practical non-identifiability can be clarified. The result of this correlation analysis provides a necessary condition for experimental design for the control signals in order to acquire suitable measurement data for unique parameter estimation. However, this method can only identify parameter correlations in *fully* observed models, i.e. it is required that all state variables be measurable, thus leading to limited applications of this method.

In this paper, we propose a simple method which can be used for identifying pairwise and higher order parameter correlations in *partially* observed linear dynamic models. An idealized measurement (i.e. noise-free and continuous data) is assumed in this study. It means that we derive the necessary condition of identifiable systems when such data are available. Unlike previous approaches, explicit relations of the identifiability to initial condition and control functions can be found by our method, thus providing a useful guidance for experimental design for remedying the non-identifiability if available. Our basic idea is to derive the output sensitivity matrix and detect the linear dependences of its columns, which can be simply made by using the Laplace transformation and then solving a set of homogenous linear equations. The computations are quite straightforward and thus can be easily implemented into a software packet.

For parameter estimation, different species (substances) measured from experiment leads to different output equations, due to which the model parameters may be non-identifiable. Therefore, prior to the experiment for data acquisition, it is important to analyze the identifiability of the parameters, when some certain species will be measured. The method presented in this paper can be used for *a priori* identifiability analysis. In this way, the question which species should be measured, so that unique parameter estimation will be achieved, can be answered. Thus, this is important for experimental design.

Both structural and practical non-identifiability can be addressed by using the proposed method. In the case of structural non-identifiability, the identifiable parameter combinations can be determined, while, in the case of practical non-identifiability, this method can provide suggestions for experimental design (i.e. initial condition, constant control signals, and noise-free datasets) so as to remedy the non-identifiability. Five linear compartmental models including an insulin receptor dynamics model are taken to demonstrate the effectiveness of our approach.

## Methods

### Output sensitivity matrix

In this paper, we consider general time-invariant linear state space models described as

1 $$ \dot{\mathbf{x}}=\mathbf{Ax}+\mathbf{B}\mathbf{u},\kern1em \mathbf{x}(0)={\mathbf{x}}_0 $$

2 $$ \mathbf{y}=\mathbf{C}\mathbf{x}+\mathbf{D}\mathbf{u} $$

where Eq. (1) and Eq. (2) are state and output equations in which $\mathbf{x}\in {R}^{n_x},\mathbf{u}\in {R}^{n_u},\mathbf{y}\in {R}^{n_y}$ are state, control and output vectors, respectively. **x**_0_ is the initial state vector. For the purpose of parameter estimation, the controls **u** and the initial condition **x**_0_ are regarded as being defined by experimental design.

The outputs **y** are considered as variables with available datasets (time courses) measured from experiment. In many situations, due to limiting experimental facility, only part of the state variables can be measured, i.e. *n*_*y*_ < *n*_*x*_, which means that the model is partially observed. If, in the particular situation, *n*_*y*_ = *n*_*x*_, the model is called fully observed. Similarly, the initial state vector **x**_0_ may also be partially or fully observed, depending on the measurement facility. In the following, it will be seen that the impact of the availability of partial or full observations of **x** and/or **x**_0_ are significant on the parameter correlations and thus the identifiability of the model under consideration.

It should be noted that, in the case of an observable system, the state profiles can be uniquely reconstructed even if *n*_*y*_ < *n*_*x*_. However, such a reconstruction can be made by different value sets of the parameters if there is a parameter correlation. This is due to the fact that the observability of a system depends only on the matrixes **A** and **C**, but the identifiability depends not only on **A** and **C** but also on the control signal and initial condition.

In Eq. (1) and Eq. (2), $\mathbf{A}\in {R}^{n_x\times {n}_x},\mathbf{B}\in {R}^{n_x\times {n}_u},\mathbf{C}\in {R}^{n_y\times {n}_x},\mathbf{D}\in {R}^{n_y\times {n}_u}$ are constant matrices with appropriate dimensions which contain *n*_*A*_, *n*_*B*_, *n*_*C*_, *n*_*D*_ parameters denoted in the vector form ${\mathbf{p}}_A\in {R}^{n_A},{\mathbf{p}}_B\in {R}^{n_B},{\mathbf{p}}_C\in {R}^{n_C},{\mathbf{p}}_D\in {R}^{n_D}$, respectively. Then the vector of the whole parameters to be estimated is

3 $$ {\mathbf{p}}^T=\left({\mathbf{p}}_A^T,{\mathbf{p}}_B^T,{\mathbf{p}}_C^T,{\mathbf{p}}_D^T\right) $$

It is noted that the corresponding vectors in **p**^*T*^ will fall out, when one or more matrices among (**A**, **B**, **C**, **D**) contains no parameters to be estimated. To estimate the model parameters, one needs at first to check their identifiability. Here we address this issue by identifying correlations among the parameters. To do this, it is necessary to analyze the linear dependencies of the columns of the output sensitivity matrix

4 $$ \frac{\partial \mathbf{y}}{\partial \mathbf{p}}=\left(\frac{\partial \mathbf{y}}{\partial {\mathbf{p}}_A},\frac{\partial \mathbf{y}}{\partial {\mathbf{p}}_B},\frac{\partial \mathbf{y}}{\partial {\mathbf{p}}_C},\frac{\partial \mathbf{y}}{\partial {\mathbf{p}}_D}\right) $$

where $\frac{\partial \mathbf{y}}{\partial {\mathbf{p}}_A}\in {R}^{n_y\times {n}_A},\frac{\partial \mathbf{y}}{\partial {\mathbf{p}}_B}\in {R}^{n_y\times {n}_B},\frac{\partial \mathbf{y}}{\partial {\mathbf{p}}_C}\in {R}^{n_y\times {n}_C},\frac{\partial \mathbf{y}}{\partial {\mathbf{p}}_D}\in {R}^{n_y\times {n}_D}$ are the matrices of the output sensitivities to the parameters in (**A**, **B**, **C**, **D**), respectively. Thus the output sensitivity matrix $\frac{\partial \mathbf{y}}{\partial \mathbf{p}}$ has *n*_*y*_ rows and *n*_*p*_ = *n*_*A*_ + *n*_*B*_ + *n*_*C*_ + *n*_*D*_ columns. The necessary condition of completely identifiable parameters of the model is that the columns of this matrix are linearly independent.

Solving the state Eq. (1) in the Laplace form we have

5 $$ \mathbf{X}(s)={\left(s\mathbf{I}-\mathbf{A}\right)}^{-1}\left(\mathbf{B}\mathbf{U}(s)+{\mathbf{x}}_0\right) $$

where $\mathbf{I}\in {R}^{n_x\times {n}_x}$ is an identity matrix. It follows from the output equation Eq. (2)

6 $$ \mathbf{Y}(s)=\mathbf{C}{\left(s\mathbf{I}-\mathbf{A}\right)}^{-1}\left(\mathbf{B}\mathbf{U}(s)+{\mathbf{x}}_0\right)+\mathbf{D}\mathbf{U}(s) $$

From Eq. (6), it is straightforward to achieve the output sensitivities in the Laplace form

7 $$ \begin{array}{l}\frac{\partial \mathbf{Y}(s)}{\partial {\mathbf{p}}_A}=\mathbf{C}{\left(s\mathbf{I}-\mathbf{A}\right)}^{-1}{\mathbf{M}}_A\left(\mathbf{X}(s)\right)\\ {}\frac{\partial \mathbf{Y}(s)}{\partial {\mathbf{p}}_B}=\mathbf{C}{\left(s\mathbf{I}-\mathbf{A}\right)}^{-1}{\mathbf{M}}_B\left(\mathbf{U}(s)\right)\\ {}\frac{\partial \mathbf{Y}(s)}{\partial {\mathbf{p}}_C}={\mathbf{M}}_C\left(\mathbf{X}(s)\right)\kern1em \\ {}\frac{\partial \mathbf{Y}(s)}{\partial {\mathbf{p}}_D}={\mathbf{M}}_D\left(\mathbf{U}(s)\right)\end{array} $$

where ${\mathbf{M}}_A\left(\mathbf{X}(s)\right)=\frac{\partial }{\partial {\mathbf{p}}_A}\left(\mathbf{AX}(s)\right)$, ${\mathbf{M}}_B\left(\mathbf{U}(s)\right)=\frac{\partial }{\partial {\mathbf{p}}_B}\left(\mathbf{B}\mathbf{U}(s)\right)$, ${\mathbf{M}}_C\left(\mathbf{X}(s)\right)=\frac{\partial }{\partial {\mathbf{p}}_C}\left(\mathbf{C}\mathbf{X}(s)\right)$ and ${\mathbf{M}}_D\left(\mathbf{U}(s)\right)=\frac{\partial }{\partial {\mathbf{p}}_D}\left(\mathbf{D}\mathbf{U}(s)\right)$ are partial derivative matrices with ${\mathbf{M}}_A\in {R}^{n_x\times {n}_A}$, ${\mathbf{M}}_B\in {R}^{n_x\times {n}_B}$ , ${\mathbf{M}}_C{\in}^{n_y\times {n}_C}$ and ${\mathbf{M}}_D{\in}^{n_y\times {n}_D}$, in which the corresponding elements are linear to the elements of **X**(*s*) and **U**(*s*), respectively. Therefore, the output sensitivity matrix Eq. (4) can be written as

8 $$ \frac{\partial \mathbf{Y}(s)}{\partial \mathbf{p}}={\left(\begin{array}{c}\hfill \mathbf{C}{\left(s\mathbf{I}-\mathbf{A}\right)}^{-1}{\mathbf{M}}_A\left(\mathbf{X}(s)\right)\hfill \\ {}\hfill \mathbf{C}{\left(s\mathbf{I}-\mathbf{A}\right)}^{-1}{\mathbf{M}}_B\left(\mathbf{U}(s)\right)\hfill \\ {}\hfill {\mathbf{M}}_C\left(\mathbf{X}(s)\right)\hfill \\ {}\hfill {\mathbf{M}}_D\left(\mathbf{U}(s)\right)\hfill \end{array}\right)}^T $$

According to Eq. (5) and Eq. (7), the elements in the output sensitivity matrix Eq. (8) are functions of the parameters in the matrices **A**, **B**, **C**, **D**, the elements of the input vector **U**(*s*) and of the initial condition vector **x**_0_. In this way, explicit relationships of parameter sensitivities with the controls and the initial condition can be achieved, base on which both structural and practical identifiability issues can be addressed.

It is noted that in most previous studies on identifiability analysis of linear models only parameters in the matrix **A** were considered as being estimated. In this case, **M**_*B*_ = **0**, **M**_*C*_ = **0**, **M**_*D*_ = **0** and thus only the first term in Eq. (8) remains, i.e.

9 $$ \frac{\partial \mathbf{Y}(s)}{\partial \mathbf{p}}=\mathbf{C}{\left(s\mathbf{I}-\mathbf{A}\right)}^{-1}{\mathbf{M}}_A\left(\mathbf{X}(s)\right) $$

Moreover, in the case of **y** = **x**, then Eq. (9) reduces to

10 $$ \frac{\partial \mathbf{Y}(s)}{\partial \mathbf{p}}={\left(s\mathbf{I}-\mathbf{A}\right)}^{-1}{\mathbf{M}}_A\left(\mathbf{X}(s)\right) $$

Therefore, the parameter correlations can be determined easily by checking the linear dependencies of the columns of **M**_*A*_(**X**(*s*)). The identification of parameter correlations in the case of **y** = **x** was in detail discussed in [15].

### Identification of parameter correlations

To identify the correlations among the parameters, it is necessary to analyze the linear dependencies of the output sensitivity matrix Eq. (8) which can be expressed as (see Additional file 1)

11 $$ \frac{\partial \mathbf{Y}(s)}{\partial \mathbf{p}}=\frac{1}{\varDelta^2}\left(\begin{array}{cccc}\hfill {\mathbf{Q}}_A(s)\hfill & \hfill {\mathbf{Q}}_B(s)\hfill & \hfill {\mathbf{Q}}_C(s)\hfill & \hfill {\mathbf{Q}}_D(s)\hfill \end{array}\right) $$

where Δ = det(*s***I** − **A**) and

12 $$ \begin{array}{l}{\mathbf{Q}}_A(s)=\left(\begin{array}{ccc}\hfill {q}_{1,1}(s)\hfill & \hfill \cdots \hfill & \hfill {q}_{1,{n}_A}(s)\hfill \\ {}\hfill {q}_{2,1}(s)\hfill & \hfill \cdots \hfill & \hfill {q}_{2,{n}_A}(s)\hfill \\ {}\hfill \vdots \hfill & \hfill \vdots \hfill & \hfill \vdots \hfill \\ {}\hfill {q}_{n_y,1}(s)\hfill & \hfill \cdots \hfill & \hfill {q}_{n_y,{n}_A}(s)\hfill \end{array}\right),\kern1em {\mathbf{Q}}_B(s)=\left(\begin{array}{ccc}\hfill {q}_{1,{n}_A+1}(s)\hfill & \hfill \cdots \hfill & \hfill {q}_{1,{n}_A+{n}_B}(s)\hfill \\ {}\hfill {q}_{2,{n}_A+1}(s)\hfill & \hfill \cdots \hfill & \hfill {q}_{2,{n}_A+{n}_B}(s)\hfill \\ {}\hfill \vdots \hfill & \hfill \vdots \hfill & \hfill \vdots \hfill \\ {}\hfill {q}_{n_y,{n}_A+1}(s)\hfill & \hfill \cdots \hfill & \hfill {q}_{n_y,{n}_A+{n}_B}(s)\hfill \end{array}\right)\\ {}{\mathbf{Q}}_C(s)=\left(\begin{array}{ccc}\hfill {q}_{1,{n}_A+{n}_B+1}(s)\hfill & \hfill \cdots \hfill & \hfill {q}_{1,{n}_A+{n}_B+{n}_C}(s)\hfill \\ {}\hfill {q}_{2,{n}_A+{n}_B+1}(s)\hfill & \hfill \cdots \hfill & \hfill {q}_{2,{n}_A+{n}_B+{n}_C}(s)\hfill \\ {}\hfill \vdots \hfill & \hfill \vdots \hfill & \hfill \vdots \hfill \\ {}\hfill {q}_{n_y,{n}_A+{n}_B+1}(s)\hfill & \hfill \cdots \hfill & \hfill {q}_{n_y,{n}_A+{n}_B+{n}_C}(s)\hfill \end{array}\right),\kern1em {\mathbf{Q}}_C(s)=\left(\begin{array}{ccc}\hfill {q}_{1,{n}_A+{n}_B+{n}_C+1}(s)\hfill & \hfill \cdots \hfill & \hfill {q}_{1,{n}_p}(s)\hfill \\ {}\hfill {q}_{2,{n}_A+{n}_B+{n}_C+1}(s)\hfill & \hfill \cdots \hfill & \hfill {q}_{2,{n}_p}(s)\hfill \\ {}\hfill \vdots \hfill & \hfill \vdots \hfill & \hfill \vdots \hfill \\ {}\hfill {q}_{n_y,{n}_A+{n}_B+{n}_C+1}(s)\hfill & \hfill \cdots \hfill & \hfill {q}_{n_y,{n}_p}(s)\hfill \end{array}\right)\end{array} $$

In Eq. (12), each of the elements *q*_*i*,*j*_(*s*), (*i* = 1, ⋯, *n*_*y*_, *j* = 1, ⋯, *n*_*p*_) is a polynomial with the indeterminate *s*. As mentioned above, the coefficients of these polynomials will be functions of the parameters in **p**_*A*_, **p**_*B*_, **p**_*C*_, **p**_*D*_, the elements in the input vector **U**(*s*) and in the initial condition vector **x**_0_. It can be shown from Additional file 1 that the highest order of the polynomials in the 4 matrices in Eq. (12) will be 2(*n*_*x*_ − 1), 2*n*_*x*_ − 1, 2*n*_*x*_ − 1, 2*n*_*x*_, respectively. Based on Eq. (12), Eq. (11) can be rewritten as

13 $$ \frac{\partial \mathbf{Y}(s)}{\partial \mathbf{p}}=\frac{1}{\varDelta^2}\left(\begin{array}{cccc}\hfill {\mathbf{q}}_1(s)\hfill & \hfill {\mathbf{q}}_2(s)\hfill & \hfill \cdots \hfill & \hfill {\mathbf{q}}_{n_p}(s)\hfill \end{array}\right) $$

where ${\mathbf{q}}_j(s)\in {R}^{n_y},\kern0.5em \left(j=1,\cdots, {n}_p\right)$ are the columns of the matrices in Eq. (12). To check the linear dependencies of the columns in Eq. (13), we introduce a vector $\boldsymbol{\upalpha} ={\left({\alpha}_1,{\alpha}_2,\cdots, {\alpha}_{n_p}\right)}^T$ and let

14 $$ {\alpha}_1{\mathbf{q}}_1(s)+{\alpha}_2{\mathbf{q}}_2(s)+\cdots +{\alpha}_{n_p}{\mathbf{q}}_{n_p}(s)=\mathbf{0} $$

According to Eq. (11) and Eq. (12), Eq. (14) consists of *n*_*y*_ linear equations with respect to $\left({\alpha}_1,{\alpha}_2,\cdots, {\alpha}_{n_p}\right)$ as unknowns. Since **q**_*j*_(*s*), (*j* = 1, ⋯, *n*_*p*_) are explicitly expressed polynomials, we can reorder the terms in Eq. (14) and present it in the following polynomial form

15 $$ {\gamma}_{i,1}{s}^{2{n}_x}+{\gamma}_{i,2}{s}^{2{n}_x-1}+\cdots +{\gamma}_{i,2{n}_x}s+{\gamma}_{i,2{n}_x+1}=0,\kern1em i=1,\cdots, {n}_y $$

where the coefficients *γ*_*i*,*k*_, (*k* = 1, ⋯, 2*n*_*x*_ + 1) are linear to the elements of $\left({\alpha}_1,{\alpha}_2,\cdots, {\alpha}_{n_p}\right)$. Since *s* in Eq. (15) is indeterminate, each coefficient of the polynomials in Eq. (15) should be zero, i.e.

16 $$ {\gamma}_{i,k}={\beta}_{i,k,1}{\alpha}_1+{\beta}_{i,k,2}{\alpha}_2+\cdots +{\beta}_{i,k,{n}_p}{\alpha}_{n_p}=0 $$

where the coefficients *β*_*i*,*k*,*l*_, (*l* = 1, ⋯, *n*_*p*_) are some functions of the model parameters, of the elements in the input vector as well as in the initial state vector. Therefore, Eq. (16) represents a set of homogeneous linear equations with $\left({\alpha}_1,{\alpha}_2,\cdots, {\alpha}_{n_p}\right)$ as unknowns. The maximum number of the equations is *n*_*y*_(2*n*_*x*_ + 1).

It is to note that the highest order of the polynomials in Eq. (15) is 2*n*_*x*_ when all four matrices **A**, **B**, **C**, **D** contain parameters to be estimated. If the problem under consideration is not in this case, the highest order in Eq. (15) should vary accordingly. For instance, if the model has parameters only in matrix **A**, as described by Eq. (9), the highest order in Eq. (15) will be 2*n*_*x*_ − 2 and thus the maximum number of the equations in Eq. (16) will be *n*_*y*_(2*n*_*x*_ − 2).

In general, the solution of Eq. (16) consists of the following 2 possible cases.

1. All unknowns are zero, i.e.
  17 $$ {\alpha}_1={\alpha}_2=\cdots ={\alpha}_{n_p}=0 $$
  In this case, there is no correlation relationship among the parameters. It means that all parameters in the model are identifiable and *one* dataset is enough to uniquely estimate them.
2. A sub-group of *k* unknowns *α*_*l*+1_ ≠ 0, *α*_*l*+2_ ≠ 0,⋯, *α*_*l* + *k*_ ≠ 0 which lead to
  18 $$ {\alpha}_{l+1}{\mathbf{q}}_{l+1}(s)+{\alpha}_{l+2}{\mathbf{q}}_{l+2}(s)+\cdots +{\alpha}_{l+k}{\mathbf{q}}_{l+k}(s)=\mathbf{0} $$
  where *l* + *k* ≤ *n*_*p*_. This means that the corresponding *k* parameters (*p*_*l*+1_,⋯, *p*_*l*+*k*_) are correlated in one group. If by solving Eq. (18), **U**(*s*) and **x**_0_ are cancelled, the solutions will be independent of the controls and the initial condition. Then the corresponding parameters are *structurally* non-identifiable. This means that the parameters cannot be estimated based on any datasets.
  If a model is structurally non-identifiable, there will exist identifiable combinations of the parameters and these combinations may have one or a finite number of solutions [34, 35, 39]. Identifiable combinations are sub-groups of the correlated parameters expressing their explicit interrelationships, which can be obtained by solving the homogenous linear partial-differential equations (from Eq. (13) and Eq. (18)) based on the result of *α*_*l*+1_, *α*_*l*+2_,⋯, *α*_*l*+*k*_.
  In contrast, if the solutions of Eq. (18) depend on the controls **U**(*s*) and/or the initial condition **x**_0_, the corresponding parameters are *practically* non-identifiable. Since Eq. (18) describes the parameter correlation based on **U**(*s*) and **x**_0_ which cause a specific dataset, using (noise-free) datasets from different controls and initial conditions, Eq. (18) can be written as
  19 $$ {\alpha}_{l+1}{\mathbf{q}}_{l+1}^{(r)}(s)+{\alpha}_{l+2}{\mathbf{q}}_{l+2}^{(r)}(s)+\cdots +{\alpha}_{l+k}{\mathbf{q}}_{l+k}^{(r)}(s)=\mathbf{0} $$
  where (*r*) denotes using the dataset *r* caused by **U**^(*r*)^(*s*) and **x**_0_^(*r*)^. In this paper, for one dataset we mean the measured output profiles caused by an initial state condition and constant control signals during the experiment. If *n*_*d*_ datasets with different controls and/or initial conditions are used, i.e. *r* = 1, ⋯, *n*_*d*_, for the parameter estimation, the columns of the following matrix will be independent
  20 $$ \mathbf{Q}(s)=\left(\begin{array}{cccc}\hfill {\mathbf{q}}_{l+1}^{(1)}(s)\hfill & \hfill {\mathbf{q}}_{l+2}^{(1)}(s)\hfill & \hfill \cdots \hfill & \hfill {\mathbf{q}}_{l+k}^{(1)}(s)\hfill \\ {}\hfill {\mathbf{q}}_{l+1}^{(2)}(s)\hfill & \hfill {\mathbf{q}}_{l+2}^{(2)}(s)\hfill & \hfill \cdots \hfill & \hfill {\mathbf{q}}_{l+k}^{(2)}(s)\hfill \\ {}\hfill \vdots \hfill & \hfill \vdots \hfill & \hfill \vdots \hfill & \hfill \vdots \hfill \\ {}\hfill {\mathbf{q}}_{l+1}^{\left({n}_d\right)}(s)\hfill & \hfill {\mathbf{q}}_{l+2}^{\left({n}_d\right)}(s)\hfill & \hfill \cdots \hfill & \hfill {\mathbf{q}}_{l+k}^{\left({n}_d\right)}(s)\hfill \end{array}\right) $$
  where $\mathbf{Q}(s)\in {R}^{\left({n}_d\times {n}_y\right)\times \left(l+k\right)}$. The number of datasets *n*_*d*_ should be so selected, that the total number of equations (i.e. *n*_*d*_ times the number of equations in Eq. (19)) is greater than *k*. As a result, there will be *α*_*l*+1_ =⋯= *α*_*l*+*k*_ = 0. This means that the practical non-identifiability described as Eq. (18) is remedied. In this way, the corresponding parameters (*p*_*l*+1_, ⋯, *p*_*l*+*k*_) can be uniquely estimated.
  In Eq. (18) and Eq. (19), the parameters are pairwise correlated if *k* = 2, whereas there is a higher order interrelationship among the parameters if *k* > 2. In a model with a large number of parameters to be estimated, there may be many sub-groups with different numbers of correlated parameters. All sub-groups can be determined by solving Eq. (16). We denote *n*_max_ as the maximum number of parameters among the sub-groups. Therefore, the number of (noise-free) datasets from different control signals and initial conditions should be equal to or larger than the number of *n*_max_ divided by the number of equations in Eq. (19), in order to remedy the practical non-identifiability of the model. The impact of control signals and the initial condition on the linear dependency of the columns in Eq. (20) is implicitly given in Eqs. (5)-(7).

In summary, the method presented above can be used to identify parameter correlations related both to control signals and to the initial condition for partially observed linear systems. This is an extension of the method proposed in [15] where the necessary conditions of parameter correlations were given only based on the model structure and a full state observation. However, the application of the method of this paper is limited due to the computations using symbolic algebra for solving Eq. (14), since symbolic algebra has its limits in the size of the problem (i.e. the number of parameters) it can solve. During this study, we have solved a series of problems with a maximum size of ten parameters with a commonly used personal computer.

## Results

### Example 1

A linear two-compartment model [39, 45]

21 $$ \begin{array}{l}{\dot{x}}_1=-\left({p}_1+{p}_2\right){x}_1+{p}_3{x}_2+u,\kern1em {x}_1(0)={x}_{10}\\ {}{\dot{x}}_2={p}_2{x}_1-\left({p}_3+{p}_4\right){x}_2,\kern3.5em {x}_2(0)={x}_{20}\\ {}y={x}_1/V\end{array} $$

**Fig. 1:**
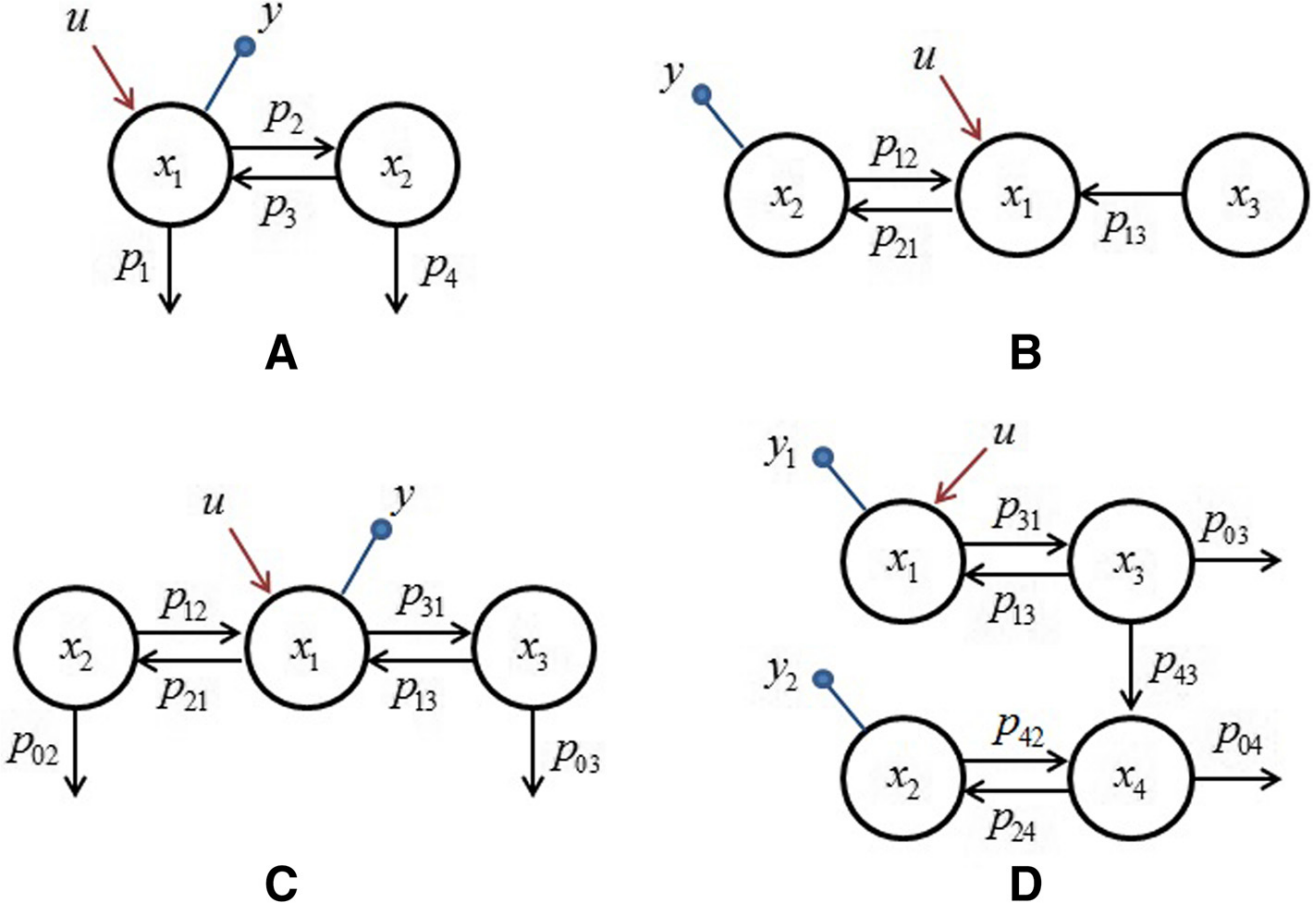
Biochemical reaction networks of the compartment models used in examples 1—4. **a**: 2-compartment model in example 1; **b**: 3-compartment model in example 2; **c**: 3-compartment model in example 3; **d**: 4-compartment model in example 4

This model describes a simple biochemical reaction network as shown in Fig. 1a. This model is partially observed and the partial observation causes structural non-identifiability. Unlike previous studies of this well-known model, the impact of the initial conditions on the identifiability is highlighted here. In this example, there are 5 parameters, i.e. **p**_*A*_ = (*p*_1_, *p*_2_, *p*_3_, *p*_4_)^*T*^, **p**_*C*_ = *V* and then we have

22 $$ {\mathbf{M}}_A\left(\mathbf{X}(s)\right)=\left(\begin{array}{cccc}\hfill -{X}_1(s)\hfill & \hfill -{X}_1(s)\hfill & \hfill {X}_2(s)\hfill & \hfill 0\hfill \\ {}\hfill 0\hfill & \hfill {X}_1(s)\hfill & \hfill -{X}_2(s)\hfill & \hfill -{X}_2(s)\hfill \end{array}\right),\kern0.5em {\mathbf{M}}_C\left(\mathbf{X}(s)\right)=-\frac{1}{V^2}{X}_1(s) $$

The Laplace form of the state variables can be achieved by solving the state equations in Eq. (21)

23 $$ \begin{array}{l}{X}_1(s)=\frac{1}{\varDelta}\left(\left(s+{p}_3+{p}_4\right)\left(U(s)+{x}_{10}\right)+{p}_3{x}_{20}\right)\\ {}{X}_2(s)=\frac{1}{\varDelta}\left({p}_2\left(U(s)+{x}_{10}\right)+\left(s+{p}_1+{p}_2\right){x}_{20}\right)\end{array} $$

where Δ = (*s* + *p*_1_ + *p*_2_)(*s* + *p*_3_ + *p*_4_) − *p*_2_*p*_3_. According to Eq. (13), the output sensitivity vector (not a matrix, since there is only one output variable in this example) is expressed as

24 $$ \frac{\partial Y(s)}{\partial \mathbf{p}}=\frac{-1}{V\varDelta }{\left(\begin{array}{c}\hfill \left(s+{p}_3+{p}_4\right){X}_1(s)\hfill \\ {}\hfill \left(s+{p}_4\right){X}_1(s)\hfill \\ {}\hfill -\left(s+{p}_3+{p}_4\right){X}_2(s)\hfill \\ {}\hfill \begin{array}{c}\hfill {p}_3{X}_2(s)\hfill \\ {}\hfill \frac{\varDelta }{V}{X}_1(s)\hfill \end{array}\hfill \end{array}\right)}^T $$

Applying the expressions of Eq. (22) to Eq. (23), it follows

25 $$ \frac{\partial Y(s)}{\partial \mathbf{p}}=\frac{-1}{V^2{\varDelta}^2}{\left(\begin{array}{c}\hfill V\left(s+{p}_3+{p}_4\right)\left(\left(s+{p}_3+{p}_4\right)\left(U(s)+{x}_{10}\right)+{p}_3{x}_{20}\right)\hfill \\ {}\hfill V\left(s+{p}_4\right)\left(\left(s+{p}_3+{p}_4\right)\left(U(s)+{x}_{10}\right)+{p}_3{x}_{20}\right)\hfill \\ {}\hfill -V\left(s+{p}_4\right)\left({p}_2\left(U(s)+{x}_{10}\right)+\left(s+{p}_1+{p}_2\right){x}_{20}\right)\hfill \\ {}\hfill \begin{array}{c}\hfill V{p}_3\left({p}_2\left(U(s)+{x}_{10}\right)+\left(s+{p}_1+{p}_2\right){x}_{20}\right)\hfill \\ {}\hfill \left(\left(s+{p}_3+{p}_4\right)\left(U(s)+{x}_{10}\right)+{p}_3{x}_{20}\right)\varDelta \hfill \end{array}\hfill \end{array}\right)}^T $$

To analyze the linear dependencies of the 5 functions in Eq. (25) we introduce 5 unknowns (*α*_1_, ⋯, *α*_5_). By using the method described in the above section (see Additional file 1), we find *α*_5_ = 0, i.e. the parameter *V* is uncorrelated with any other parameters and thus is uniquely identifiable. This result is trivial in fact, since *V* is immediately fixed if *y*(0) and *x*_1_(0) are known, according to the last line of Eq. (21).

It can be seen from Eq. (25) that *U*(*s*) and *x*_10_ have the same impact on the output sensitivities. Thus, to see the influence of the initial conditions on the identifiability of the 4 parameters (*p*_1_, *p*_2_, *p*_3_, *p*_4_), we let *U*(*s*) = 0 in the following analysis.

1. *x*_10_ ≠ 0, *x*_20_ = 0. The resulting output sensitivity to the individual parameters has the following relationship (see Additional file 1)
  26 $$ \frac{\partial Y(s)}{\partial {p}_1}-\frac{\partial Y(s)}{\partial {p}_2}+\frac{p_3}{p_2}\frac{\partial Y(s)}{\partial {p}_3}-\frac{p_3}{p_2}\frac{\partial Y(s)}{\partial {p}_4}=0 $$
  This means that the 4 parameters are correlated in one group and their interrelationship is independent of the value of *x*_10_ ≠ 0, i.e. they are *structurally* non-identifiable. By solving Eq. (26) we can find the interrelationships of the sub-groups (also called identifiable combinations) of the parameters as {*p*_2_*p*_3_, *p*_1_ + *p*_2_, *p*_3_ + *p*_4_}. This result is the same as reported in the literature [39, 45]. In this situation, it is impossible to uniquely estimate the parameters (*p*_1_, *p*_2_, *p*_3_, *p*_4_) based on any measured datasets of the output (*y* = *x*_1_).
2. *x*_10_ = 0, *x*_20_ ≠ 0. Solving the linear homogenous linear equations (see Additional file 1) in this case leads to *α*_3_ = 0, i.e. *p*_3_ is identifiable. And the output sensitivity to the other parameters has the following relationship
  27 $$ \left({p}_4-{p}_1-{p}_2\right)\frac{\partial Y(s)}{\partial {p}_1}+\left({p}_1+{p}_2-{p}_3-{p}_4\right)\frac{\partial Y(s)}{\partial {p}_2}+{p}_3\frac{\partial Y(s)}{\partial {p}_4}=0 $$
  which means that (*p*_1_, *p*_2_, *p*_4_) are correlated in one group and thus structurally non-identifiable. It is interesting to note from Eq. (27) that the correlation relation of (*p*_1_, *p*_2_, *p*_4_) is also related to the identifiable parameter *p*_3_.
3. *x*_10_ ≠ 0, *x*_20_ ≠ 0. According to Eq. (25) and the Additional file 1, it can be seen that the functional relationship of the 4 parameters (*p*_1_, *p*_2_, *p*_3_, *p*_4_) depend on the initial condition (*x*_10_, *x*_20_) if both *x*_10_ ≠ 0 and *x*_20_ ≠ 0. In this situation, the parameters are *practically* non-identifiable. The 4 parameters are correlated in one group, i.e., *n*_max_ = 4, and the number of equations in the form of Eq. (19) is *n*_*y*_(2*n*_*x*_ − 2) = 2. Therefore, the 4 parameters can be uniquely estimated based on fitting the model to at least *n*_*d*_ = 2 datasets (such that *n*_*d*_ ≥ *n*_max_/(*n*_*y*_(2*n*_*x*_ − 2))) of the output (*y* = *x*_1_) from different initial values of *x*_10_ ≠ 0 and *x*_20_ ≠ 0, respectively.

**Fig. 2:**
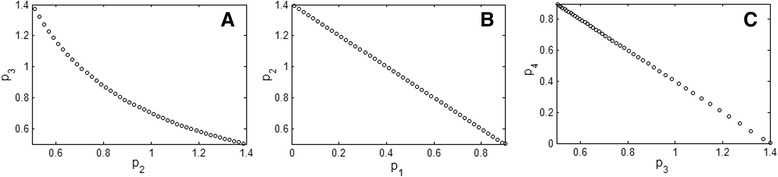
Estimation results of example 1. The identifiable combinations of the parameters are validated by repeatedly fitting the model to one dataset from the initial condition *x*_10_ ≠ 0, *x*_20_ = 0. The curves are from the results of 90 runs each of which with a different fixed value of *p*_1_. **a**: *p*_2_ *p*_3_ = 0.7; **b**: *p*_1_ + *p*_2_ = 1.4; **c**: *p*_3_ + *p*_4_ = 1.4

To verify the above achieved results, we perform numerical parameter estimation by using the method developed in [46–48]. The true parameter values in the model are assumed to be *p*_1_ = 0.7, *p*_2_ = 0.7, *p*_3_ = 1.0, *p*_4_ = 0.4 and we generate noise-free output data at 100 time points by simulation. For case 1, one dataset for *y* is generated by *x*_10_ = 15, *x*_20_ = 0. To check the identifiable combinations {*p*_2_*p*_3_, *p*_1_ + *p*_2_, *p*_3_ + *p*_4_}, we repeat the parameter identification run by fixing *p*_1_ with a different value for each run. Figure 2 shows the relationships of the parameters after the fitting which illustrate exactly the expected function values, i.e. *p*_2_*p*_3_ = 0.7, *p*_1_ + *p*_2_ = 1.4, *p*_3_ + *p*_4_ = 1.4.

For case 2, one dataset for *y* is generated by *x*_10_ = 0, *x*_20_ = 15. Indeed, only the true value of *p*_3_ = 1.0 can be identified when fitting the 4 parameters to the dataset. For case 3, we generate 2 datasets for *y* by *x*_10_^(1)^ = 15, *x*_20_^(2)^ = 5 and *x*_10_^(2)^ = 5, *x*_20_^(2)^ = 15, respectively. Then we fit the 4 parameters simultaneously to the 2 datasets, from which we obtain the estimated values of the parameters exactly as their true values.

Furthermore, if the model in Eq. (21) is fully observed, e.g., *y*_1_ = *x*_1_, *y*_2_ = *x*_2_, then all parameters in the model are identifiable. This can be easily seen from **M**_*A*_(**X**(*s*)) in Eq. (22), since its columns are linearly independent. As a result, one single dataset including the trajectories of *y*_1_ = *x*_1_, *y*_2_ = *x*_2_ is enough to uniquely estimate the parameters in the model.

### Example 2

A linear three-compartment model [43]

28 $$ \begin{array}{l}{\dot{x}}_1={p}_{13}{x}_3+{p}_{12}{x}_2-{p}_{21}{x}_1+u,\kern1em {x}_1(0)={x}_{10}\\ {}{\dot{x}}_2={p}_{21}{x}_1-{p}_{12}{x}_2,\kern5em {x}_2(0)={x}_{20}\\ {}{\dot{x}}_3=-{p}_{13}{x}_3,\kern7.12em {x}_3(0)={x}_{30}\\ {}y={x}_2\end{array} $$

The reaction network corresponding to this model is shown in Fig. 1b. This model was studied in [43] to demonstrate the failure of the identifiability test by using the differential algebra methods when *x*_30_ = 0. This can be easily recognized by using our method. Let **p**_*A*_ = (*p*_21_, *p*_12_, *p*_13_)^*T*^, according to Eq. (13), the functions in the output sensitivity vector will be (see Additional file 1).

29 $$ \begin{array}{l}{q}_1(s)=\left(s+{p}_{13}\right)\left(\left(U(s)+{x}_{10}\right){s}^2+\left(\left({p}_{12}+{p}_{13}\right)\left(U(s)+{x}_{10}\right)+{p}_{12}{x}_{20}+{p}_{13}{x}_{30}\right)s+{p}_{12}{p}_{13}\left(U(s)+{x}_{10}+{x}_{20}+{x}_{30}\right)\right)\\ {}{q}_2(s)=-\left(s+{p}_{13}\right)\left({x}_{20}{s}^2+\left(\left({p}_{21}+{p}_{13}\right){x}_{20}+{p}_{21}\left(U(s)+{x}_{10}\right)\right)s+{p}_{13}{k}_{21}\left(U(s)+{x}_{10}+{x}_{20}+{x}_{30}\right)\right)\\ {}{q}_3(s)={p}_{21}{x}_{30}\left(s+{p}_{12}+{p}_{21}\right)s\end{array} $$

According to Eq. (14) and Eq. (15) we obtain the following 4 homogeneous linear equations (see Additional file 1)

30 $$ \begin{array}{l}\left(U(s)+{x}_{10}\right){\alpha}_1-{x}_{20}{\alpha}_2=0\\ {}\left(\left({p}_{12}+2{k}_{13}\right)\left(U(s)+{x}_{10}\right)+{p}_{12}{x}_{20}+{p}_{13}{x}_{30}\right){\alpha}_1-\left(\left({p}_{21}+2{p}_{13}\right){x}_{20}+{p}_{21}\left(U(s)+{x}_{10}\right)\right){\alpha}_2+{p}_{21}{x}_{30}{\alpha}_3=0\\ {}\left(\begin{array}{l}\left({p}_{13}\left(\left({p}_{12}+{p}_{13}\right)\left(U(s)+{x}_{10}\right)+{p}_{12}{x}_{20}+{p}_{13}{x}_{30}\right)+{p}_{13}{p}_{12}\left(U(s)+{x}_{10}+{x}_{20}+{x}_{30}\right)\right){\alpha}_1\\ {}-\left({p}_{13}\left(\left({p}_{21}+{p}_{13}\right){x}_{20}+{p}_{21}\left(U(s)+{x}_{10}\right)\right)+{p}_{13}{p}_{21}\left(U(s)+{x}_{10}+{x}_{20}+{x}_{30}\right)\right){\alpha}_2\\ {}+{p}_{21}\left({p}_{12}+{p}_{21}\right){x}_{30}{\alpha}_3\end{array}\right)=0\\ {}{p}_{12}{\alpha}_1-{p}_{21}{\alpha}_2=0\end{array} $$

It can be easily seen that if *x*_30_ = 0, then *α*_3_ will disappear from Eq. (30), i.e. *α*_3_ can be any value, which means that *p*_13_ is non-identifiable. On the contrary, if *x*_30_ ≠ 0, we have in Eq. (30) 4 linearly independent homogeneous equations with 3 unknowns and thus there should be *α*_1_ = *α*_2_ = *α*_3_ = 0, which means that all three parameters are identifiable.

### Example 3

A linear three-compartment model [39]

31 $$ \begin{array}{l}{\dot{x}}_1=-\left({p}_{21}+{p}_{31}\right){x}_1+{p}_{12}{x}_2+{p}_{13}{x}_3+u,\kern1em {x}_1(0)={x}_{10}\\ {}{\dot{x}}_2={p}_{21}{x}_1-\left({p}_{12}+{p}_{02}\right){x}_2,\kern6em {x}_2(0)={x}_{20}\\ {}{\dot{x}}_3={p}_{31}{x}_1-\left({p}_{13}+{p}_{03}\right){x}_3,\kern6.12em {x}_3(0)={x}_{30}\\ {}y={x}_1\end{array} $$

The reaction network described by this model is shown in Fig. 1c. The parameters to be estimated in this model are **p**_*A*_ = (*p*_21_, *p*_31_, *p*_12_, *p*_13_, *p*_02_, *p*_03_)^*T*^. The solution of the state equations in Eq. (31) leads to

32 $$ \left(\begin{array}{c}\hfill {X}_1(s)\hfill \\ {}\hfill {X}_2(s)\hfill \\ {}\hfill {X}_3(s)\hfill \end{array}\right)={\left(\begin{array}{ccc}\hfill s+\left({p}_{21}+{p}_{31}\right)\hfill & \hfill -{p}_{12}\hfill & \hfill -{p}_{13}\hfill \\ {}\hfill -{p}_{21}\hfill & \hfill s+\left({p}_{12}+{p}_{02}\right)\hfill & \hfill 0\hfill \\ {}\hfill -{p}_{31}\hfill & \hfill 0\hfill & \hfill s+\left({p}_{13}+{p}_{03}\right)\hfill \end{array}\right)}^{-1}\left(\begin{array}{c}\hfill U(s)+{x}_{10}\hfill \\ {}\hfill {x}_{20}\hfill \\ {}\hfill {x}_{30}\hfill \end{array}\right) $$

Similar to Example 2 we can obtain the Laplace functions in the output sensitivity vector as follows

33 $$ \begin{array}{l}{q}_1(s)=-\left(s+{p}_{13}+{p}_{03}\right)\left(s+{p}_{02}\right){X}_1(s)\\ {}{q}_2(s)=-\left(s+{p}_{12}+{p}_{02}\right)\left(s+{p}_{03}\right){X}_1(s)\\ {}{q}_3(s)=\left(s+{p}_{13}+{p}_{03}\right)\left(s+{p}_{02}\right){X}_2(s)\\ {}{q}_4(s)=\left(s+{p}_{12}+{p}_{02}\right)\left(s+{p}_{03}\right){X}_3(s)\\ {}{q}_5(s)=-{k}_{21}\left(s+{p}_{13}+{p}_{03}\right){X}_2(s)\\ {}{q}_6(s)=-{k}_{13}\left(s+{p}_{12}+{p}_{02}\right){X}_3(s)\end{array} $$

By introducing 6 unknowns (*α*_1_, ⋯, *α*_6_), the dependencies of the output sensitivities on the control and the initial condition can be derived from Eq. (32) and Eq. (33). If *x*_20_ = *x*_30_ = 0 and *U*(*s*) + *x*_10_ ≠ 0, the resulting homogeneous linear equations in the form of Eq. (16) are as follows

34 $$ \begin{array}{l}{\alpha}_1+{\alpha}_2=0\\ {}\left(2a+b+{p}_{02}\right){\alpha}_1+\left(2b+a+{p}_{03}\right){\alpha}_2+{p}_{21}{\alpha}_3+{p}_{31}{\alpha}_4=0\\ {}\left(\begin{array}{l}\left({a}^2+2a\left(b+{p}_{02}\right)+b{p}_{02}\right){\alpha}_1+\left({b}^2+2b\left(a+{p}_{03}\right)+a{p}_{03}\right){\alpha}_2\\ {}+{p}_{21}\left(2a+{p}_{02}\right){\alpha}_3+{p}_{31}\left(2b+{p}_{03}\right){\alpha}_4+{p}_{21}{p}_{12}{\alpha}_5+{p}_{31}{p}_{13}{\alpha}_6\end{array}\right)=0\\ {}\left(\begin{array}{l}\left({a}^2\left(b+{p}_{02}\right)+2 ab{p}_{02}\right){\alpha}_1+\left({b}^2\left(a+{p}_{03}\right)+2 ab{p}_{03}\right){\alpha}_2+{p}_{21}\left({a}^2+2a{p}_{02}\right){\alpha}_3\\ {}+{p}_{31}\left({b}^2+2b{p}_{03}\right){\alpha}_4+2a{p}_{21}{p}_{12}{\alpha}_5+2b{p}_{31}{p}_{13}{\alpha}_6\end{array}\right)=0\\ {}{a}^2b{p}_{02}{\alpha}_1+{b}^2a{p}_{03}{\alpha}_2+{k}_{21}{a}^2{p}_{02}{\alpha}_3+{p}_{31}{b}^2{p}_{03}{\alpha}_4+{p}_{21}{p}_{12}{a}^2{\alpha}_5+{p}_{31}{p}_{13}{b}^2{\alpha}_6=0\end{array} $$

where *a* = *p*_13_ + *p*_03_, *b* = *p*_12_ + *p*_02_. It can be seen that *U*(*s*) + *x*_10_ ≠ 0 does not appear in Eq. (34). Solving the 5 equations for the 6 unknowns in Eq. (34) with respect to *α*_1_, we find

35 $$ {\alpha}_2=-{\alpha}_1,\kern1em {\alpha}_3=\frac{p_{13}}{p_{31}}{\alpha}_1,\kern1em {\alpha}_4=-\frac{p_{13}}{p_{31}}{\alpha}_1,\kern1em {\alpha}_5=-\frac{p_{12}}{p_{21}}{\alpha}_1,\kern1em {\alpha}_6=\frac{p_{12}}{p_{21}}{\alpha}_1 $$

**Fig. 3:**
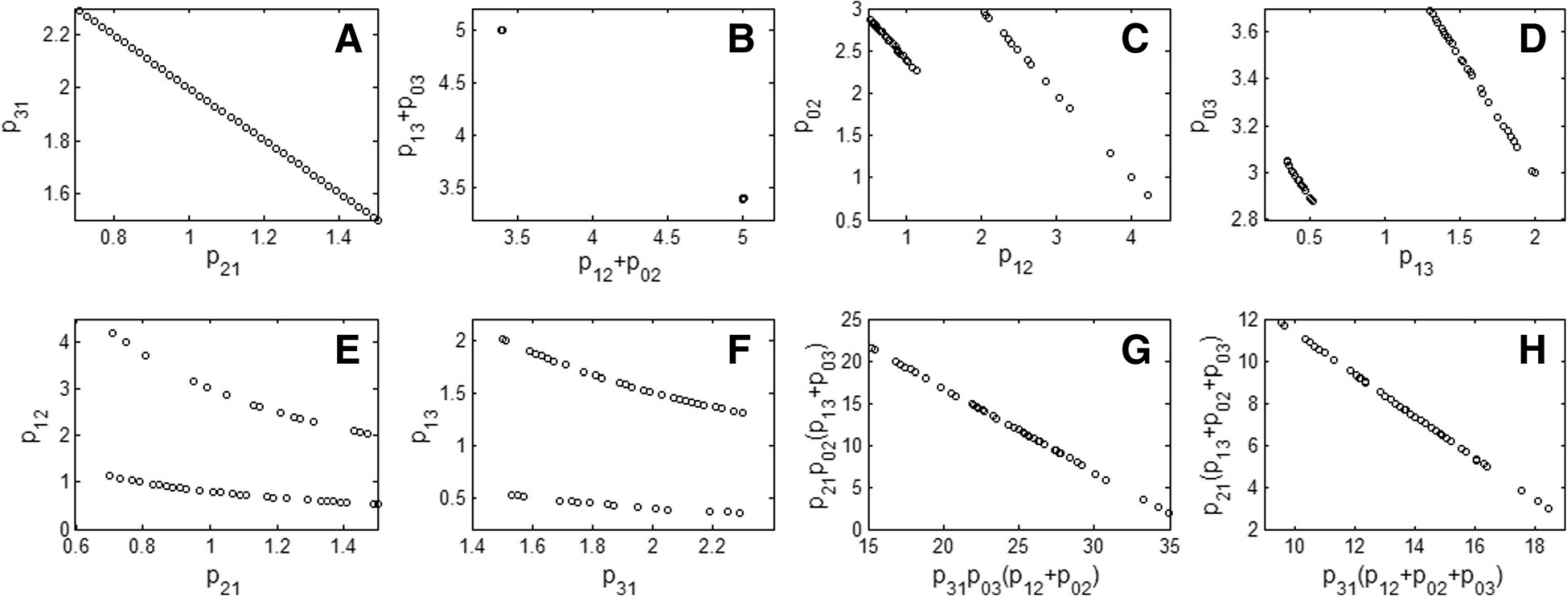
Estimation results of example 3. The identifiable combinations of the parameters are validated by repeatedly fitting the model to one dataset from *x*_10_ = *x*_20_ = *x*_30_ = 0, *u*(*t*) = 25. The curves are from the results of 82 runs each of which with a different fixed value of *p*_21_. **a**: *p*_21_ + *p*_31_ = 3.0; **b**: The relationship between *p*_02_ + *p*_12_ and *p*_03_ + *p*_13_; **c**: The relationship between *p*_02_ and *p*_12_; **d**: The relationship between *p*_03_ and *p*_13_; **e**: The relationship between *p*_12_ and *p*_21_; **f**: The relationship between *p*_13_ and *p*_31_; **g**: *p*_03_ *p*_31_(*p*_02_ + *p*_12_) + *p*_02_ *p*_21_(*p*_03_ + *p*_13_) = 36.8; **h**: *p*_31_(*p*_02_ + *p*_12_ + *p*_03_) + *p*_21_(*p*_03_ + *p*_13_ + *p*_02_) = 21.4

Thus the output sensitivities to the parameters has the following relation

36 $$ \frac{\partial Y(s)}{\partial {p}_{21}}-\frac{\partial Y(s)}{\partial {p}_{31}}+\frac{p_{13}}{p_{31}}\left(\frac{\partial Y(s)}{\partial {p}_{13}}-\frac{\partial Y(s)}{\partial {p}_{03}}\right)-\frac{p_{12}}{p_{21}}\left(\frac{\partial Y(s)}{\partial {k}_{12}}-\frac{\partial Y(s)}{\partial {k}_{02}}\right)=0 $$

which means that all 6 parameters are correlated in one group and their interrelationship is independent of *U*(*s*) + *x*_10_ ≠ 0. Thus the parameters in this model are structurally non-identifiable when *x*_20_ = *x*_30_ = 0. In addition, we can solve Eq. (36) to obtain its local solutions as {*p*_02_ + *p*_12_, *p*_03_ + *p*_13_, *p*_21_ + *p*_31_, *p*_12_*p*_21_, *p*_13_*p*_31_} as well as its global solutions as

37 $$ \begin{array}{l}{\varphi}_1={p}_{21}+{p}_{31}\\ {}{\varphi}_2=\left({p}_{02}+{p}_{12}\right)\left({p}_{03}+{p}_{13}\right)\\ {}{\varphi}_3=\left({p}_{02}+{p}_{12}\right)+\left({p}_{03}+{p}_{13}\right)\\ {}{\varphi}_4={p}_{03}{p}_{31}\left({p}_{02}+{p}_{12}\right)+{p}_{02}{p}_{21}\left({p}_{03}+{p}_{13}\right)\\ {}{\varphi}_5={p}_{31}\left({p}_{02}+{p}_{12}+{p}_{03}\right)+{p}_{21}\left({p}_{03}+{p}_{13}+{p}_{02}\right)\end{array} $$

which are the identifiable combinations of the parameters, as given in [39]. To verify these parameter relations, numerical parameter estimation is carried out by assuming *p*_02_ = 2, *p*_12_ = 3, *p*_03_ = 3, *p*_13_ = 0.4, *p*_21_ = 1, *p*_31_ = 2 as the true values of the parameters. A dataset for *y* containing 400 sampling points in the time period [0, 2] is generated by *x*_10_ = *x*_20_ = *x*_30_ = 0, *u*(*t*) = 25. Then we repeatedly fit the parameters to the dataset, except for *p*_21_ which is fixed with different values in the range [0.7, 1.5]. The estimation results are shown in Fig. 3, exactly validating the global solutions expressed as Eq. (37).

It can be seen from Fig. 3a that the relationship between *p*_21_ and *p*_31_ is indeed a straight line, namely *p*_21_ + *p*_31_ = 3.0. From Fig. 3b, it is interesting to see that the relationship between *p*_02_ + *p*_12_ and *p*_03_ + *p*_13_ is shown by two separate points. This is because both their summation and their product are constant, as indicated in *φ*_2_, *φ*_3_ of Eq. (37). This special property leads to the fact that both *p*_02_ + *p*_12_ and *p*_03_ + *p*_13_ have two solutions, i.e., there are two lines to represent the relationship of *p*_02_ + *p*_12_ as well as *p*_03_ + *p*_13_, respectively, as shown in Fig. 3c and d. Correspondingly, the relationship of *p*_12_*p*_21_as well as *p*_13_*p*_31_ is also twofold, as shown in Fig. 3e and f, respectively. The estimated results corresponding to the last two identifiable combinations in Eq. (37) are shown in Fig. 3g and h.

To remedy the non-identifiability, let *x*_20_ ≠ 0, *x*_30_ ≠ 0, then similar to Eq. (34), there will be 5 linear equations with respect to (*α*_1_, ⋯, *α*_6_). This means that the 6 parameters are correlated in one group, i.e., *n*_max_ = 6. Since the correlation relationships now depend on *U*(*s*) + *x*_10_ ≠ 0 and *x*_20_ ≠ 0, *x*_30_ ≠ 0, the parameters are practically identifiable. The number of equations in the form of Eq. (19) is *n*_*y*_(2*n*_*x*_ − 2) = 4. As a result, 2 datasets with different values of *U*(*s*) + *x*_10_ ≠ 0 and *x*_20_ ≠ 0, *x*_30_ ≠ 0 are needed to uniquely estimate the parameters of the model.

### Example 4

A linear four-compartment model [33, 39]

38 $$ \begin{array}{l}{\dot{x}}_1=-{p}_{31}{x}_1+{p}_{13}{x}_3+u,\kern5.2em {x}_1(0)={x}_{10}\\ {}{\dot{x}}_2=-{p}_{42}{x}_2+{p}_{24}{x}_4,\kern6.30em {x}_2(0)={x}_{20}\\ {}{\dot{x}}_3={p}_{31}{x}_1-\left({p}_{03}+{p}_{13}+{p}_{43}\right){x}_3,\kern2em {x}_3(0)={x}_{30}\\ {}{\dot{x}}_4={p}_{42}{x}_2+{p}_{43}{x}_3-\left({p}_{04}+{p}_{24}\right){x}_4,\kern1em {x}_4(0)={x}_{40}\\ {}{y}_1={x}_1\\ {}{y}_2={x}_2\end{array} $$

Figure 1d shows the biochemical reaction network of this model. In this model, there are 7 parameters **p**_*A*_ = (*p*_31_, *p*_13_, *p*_42_, *p*_24_, *p*_43_, *p*_03_, *p*_04_)^*T*^ and 4 state variables among which two are output variables. In the case of *u* ≠ 0, *x*_10_ = *x*_20_ = *x*_30_ = *x*_40_ = 0, the parameters are structurally non-identifiable where the identifiable combinations of the correlated parameters are expressed as {*p*_31_, *p*_13_, *p*_04_*p*_42_, *p*_24_*p*_43_, *p*_03_ + *p*_43_, *p*_24_ + *p*_42_ + *p*_04_} [33, 39]. The same results of the identifiable combinations are obtained by using our method which is much simpler to deal with, as described in the following.

From Eq. (38), we have

39 $$ \mathbf{A}=\left(\begin{array}{cccc}\hfill -{p}_{31}\hfill & \hfill 0\hfill & \hfill {p}_{13}\hfill & \hfill 0\hfill \\ {}\hfill 0\hfill & \hfill -{p}_{42}\hfill & \hfill 0\hfill & \hfill {p}_{24}\hfill \\ {}\hfill {p}_{31}\hfill & \hfill 0\hfill & \hfill -\left({p}_{03}+{p}_{13}+{p}_{43}\right)\hfill & \hfill 0\hfill \\ {}\hfill 0\hfill & \hfill {p}_{42}\hfill & \hfill {p}_{43}\hfill & \hfill -\left({p}_{04}+{p}_{24}\right)\hfill \end{array}\right),\kern1em \mathbf{B}=\left(\begin{array}{c}\hfill 1\hfill \\ {}\hfill 0\hfill \\ {}\hfill 0\hfill \\ {}\hfill 0\hfill \end{array}\right),\kern1em \mathbf{C}=\left(\begin{array}{cccc}\hfill 1\hfill & \hfill 0\hfill & \hfill 0\hfill & \hfill 0\hfill \\ {}\hfill 0\hfill & \hfill 1\hfill & \hfill 0\hfill & \hfill 0\hfill \end{array}\right) $$

and

40 $$ {\mathbf{M}}_A\left(\mathbf{X}(s)\right)=\left(\begin{array}{ccccccc}\hfill -{X}_1(s)\hfill & \hfill {X}_3(s)\hfill & \hfill 0\hfill & \hfill 0\hfill & \hfill 0\hfill & \hfill 0\hfill & \hfill 0\hfill \\ {}\hfill 0\hfill & \hfill 0\hfill & \hfill -{X}_2(s)\hfill & \hfill {X}_4(s)\hfill & \hfill 0\hfill & \hfill 0\hfill & \hfill 0\hfill \\ {}\hfill {X}_1(s)\hfill & \hfill -{X}_3(s)\hfill & \hfill 0\hfill & \hfill 0\hfill & \hfill -{X}_3(s)\hfill & \hfill -{X}_3(s)\hfill & \hfill 0\hfill \\ {}\hfill 0\hfill & \hfill 0\hfill & \hfill {X}_2(s)\hfill & \hfill -{X}_4(s)\hfill & \hfill {X}_3(s)\hfill & \hfill 0\hfill & \hfill -{X}_4(s)\hfill \end{array}\right) $$

Since in this example we have two output variables, to determine the parameter correlations we have to consider

41 $$ {\alpha}_1\left(\begin{array}{c}\hfill \frac{\partial {Y}_1}{\partial {p}_{31}}\hfill \\ {}\hfill \frac{\partial {Y}_2}{\partial {p}_{31}}\hfill \end{array}\right)+{\alpha}_2\left(\begin{array}{c}\hfill \frac{\partial {Y}_1}{\partial {p}_{13}}\hfill \\ {}\hfill \frac{\partial {Y}_2}{\partial {p}_{13}}\hfill \end{array}\right)+{\alpha}_3\left(\begin{array}{c}\hfill \frac{\partial {Y}_1}{\partial {p}_{42}}\hfill \\ {}\hfill \frac{\partial {Y}_2}{\partial {p}_{42}}\hfill \end{array}\right)+{\alpha}_4\left(\begin{array}{c}\hfill \frac{\partial {Y}_1}{\partial {p}_{24}}\hfill \\ {}\hfill \frac{\partial {Y}_2}{\partial {p}_{24}}\hfill \end{array}\right)+{\alpha}_5\left(\begin{array}{c}\hfill \frac{\partial {Y}_1}{\partial {p}_{43}}\hfill \\ {}\hfill \frac{\partial {Y}_2}{\partial {p}_{43}}\hfill \end{array}\right)+{\alpha}_6\left(\begin{array}{c}\hfill \frac{\partial {Y}_1}{\partial {p}_{03}}\hfill \\ {}\hfill \frac{\partial {Y}_2}{\partial {p}_{03}}\hfill \end{array}\right)+{\alpha}_7\left(\begin{array}{c}\hfill \frac{\partial {Y}_1}{\partial {p}_{04}}\hfill \\ {}\hfill \frac{\partial {Y}_2}{\partial {p}_{04}}\hfill \end{array}\right)=\left(\begin{array}{c}\hfill 0\hfill \\ {}\hfill 0\hfill \end{array}\right) $$

where the output sensitivities are expressed in the following form (see Additional file 1)

42 $$ \frac{\partial \mathbf{Y}(s)}{\partial \mathbf{p}}=\frac{1}{\varDelta}\left(\begin{array}{ccccccc}\hfill -\left({b}_{11}-{b}_{13}\right){X}_1\hfill & \hfill \left({b}_{11}-{b}_{13}\right){X}_3\hfill & \hfill 0\hfill & \hfill 0\hfill & \hfill -{b}_{13}{X}_3\hfill & \hfill -{b}_{13}{X}_3\hfill & \hfill 0\hfill \\ {}\hfill -\left({b}_{21}-{b}_{23}\right){X}_1\hfill & \hfill \left({b}_{21}-{b}_{23}\right){X}_3\hfill & \hfill -\left({b}_{22}-{b}_{24}\right){X}_2\hfill & \hfill \left({b}_{22}-{b}_{24}\right){X}_4\hfill & \hfill \left({b}_{24}-{b}_{23}\right){X}_3\hfill & \hfill -{b}_{23}{X}_3\hfill & \hfill -{b}_{24}{X}_4\hfill \end{array}\right) $$

It can be clearly seen from the first two columns of Eq. (41) and Eq. (42) that *α*_1_ = *α*_2_ = 0, which means that *p*_31_, *p*_13_ are uniquely identifiable. From the 5^th^ and 6^th^ columns of the first row in Eq. (42) we have *α*_5_ + *α*_6_ = 0 , i.e.

43 $$ \frac{\partial {Y}_1}{\partial {p}_{43}}-\frac{\partial {Y}_1}{\partial {p}_{03}}=0 $$

Thus *p*_43_, *p*_03_ are pairwise correlated. Furthermore, based on the second row of Eq. (41) and Eq. (42) the following results can be obtained (see Additional file 1):

44 $$ {p}_{24}\frac{\partial {Y}_2}{\partial {p}_{24}}-{p}_{43}\frac{\partial {Y}_2}{\partial {p}_{43}}=0 $$

45 $$ {p}_{42}\left(\frac{\partial {Y}_2}{\partial {p}_{42}}-\frac{\partial {Y}_2}{\partial {p}_{24}}\right)-{p}_{04}\left(\frac{\partial {Y}_2}{\partial {p}_{04}}-\frac{\partial {Y}_2}{\partial {p}_{24}}\right)=0 $$

Eq. (43)–Eq. (45) indicate that there exist 3 separate correlation groups in this example. The maximum number of parameters among the groups is 3. The (local) solutions of these 3 equations lead to the identifiable combinations of the parameters in the form of *p*_43_ + *p*_03_, *p*_24_*p*_43_, *p*_42_*p*_04_ and *p*_42_ + *p*_04_ + *p*_24_, respectively, beside the two identifiable parameters *p*_31_, *p*_13_.

**Fig. 4:**
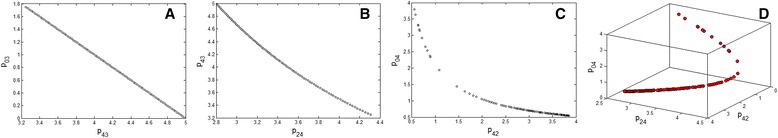
Estimation results of example 4. The identifiable combinations of the parameters are validated by repeatedly fitting the model to one dataset from *x*_10_ = *x*_20_ = *x*_30_ = *x*_40_ = 0 and *u* ≠ 0. The curves are from the results of 176 runs each of which with a different fixed value of *p*_43_. **a**: *p*_43_ + *p*_03_ = 5.0; **b**: *p*_24_ *p*_43_ = 14.0; **c**: *p*_42_ *p*_04_ = 2.1; **d**: *p*_42_ + *p*_04_ + *p*_24_ = 7.2

To numerically verify the results, *p*_31_ = 3.0, *p*_13_ = 5.5, *p*_03_ = 1.0, *p*_04_ = 0.7, *p*_24_ = 3.5, *p*_42_ = 3.0, *p*_43_ = 4.0 are used as the true values of the parameters. One noise-free dataset for *y*_1_, *y*_2_ is generated by *x*_10_ = *x*_20_ = *x*_30_ = *x*_40_ = 0 and *u* = 5.0 through simulation. Then we repeatedly fit the parameters to the dataset, except for *p*_43_ which is fixed with a different value for each run of the fitting. As expected, *p*_31_, *p*_13_ are always at their true values after each run, whereas the other parameters have correlated relationships in the forms of their identifiable combinations. Figure 4 shows these relationships based on the results of 176 runs for the parameter estimation. It can be seen from Fig. 4a to d that, indeed, *p*_43_ + *p*_03_ = 5.0, *p*_24_*p*_43_ = 14.0, *p*_42_*p*_04_ = 2.1, and *p*_42_ + *p*_04_ + *p*_24_ = 7.2 are obtained by the parameter estimation.

Also in this example, we can consider *x*_20_ ≠ 0, *x*_30_ ≠ 0, *x*_40_ ≠ 0 for remedying the non-identifiability. Since the maximum number of correlated parameter groups is *n*_max_ = 3, the number of equations in the form of Eq. (19) is *n*_*y*_(2*n*_*x*_ − 2) = 12. Therefore, one dataset for *y*_1_, *y*_2_ from an initial condition *x*_10_ ≠ 0, *x*_20_ ≠ 0, *x*_30_ ≠ 0, *x*_40_ ≠ 0 will be enough to uniquely estimate the 7 parameters in the model.

### Example 5

Insulin receptor dynamics model [49].

Many physiological processes such as glucose uptake, lipid-, protein- and glycogen-synthesis, to name the most important, are regulated by insulin after binding to the insulin receptor. The latter is located in the cytoplasma membrane [50, 51]. The insulin receptor (IR) is a dynamic cellular macromolecule. Upon insulin binding, a series of processes follow, including endocytosis of the IR-insulin complex, endosomal processing, sequestration of ligand (insulin) from the receptor, receptor inactivation as well as receptor recycling to the cell surface [52].

In several early studies, simple models describing insulin receptor dynamics were proposed [53–57] where either a subset of the whole process was considered or a few subunits were lumped into single reaction steps. More detailed models of insulin signaling pathways were developed and simulation studies were performed in [58–60]. However, parameter values such as rate constants in these models were partially taken from literature and partially estimated through experimental data.

A general five-compartment IR dynamics model was developed in [49] and its parameters were estimated based on simultaneously fitting to the measured datasets published in [55, 61, 62]. This model describes the endosomal trafficking dynamics of hepatic insulin receptor consisting of IR autophosphorylation after receptor binding, IR endosomal internalization and trafficking, insulin dissociation from and dephosphorylation of internalized IR, and finally recycling of the insulin-free, dephosphorylated IR to the plasma membrane [49]. The state equations of the general five-compartment model are given as follows [49]

46 $$ \begin{array}{l}{\dot{x}}_1={p}_{12}{x}_2+{p}_{15}{x}_5-\left({p}_{21}u+{p}_{51}\right){x}_1\\ {}{\dot{x}}_2={p}_{21}u\kern0.1em {x}_1-\left({p}_{12}+{p}_{32}\right){x}_2\\ {}{\dot{x}}_3={p}_{32}{x}_2-{p}_{43}{x}_3\\ {}{\dot{x}}_4={p}_{43}{x}_3-{p}_{54}{x}_4\\ {}{\dot{x}}_5={p}_{51}{x}_1+{p}_{54}{x}_4-{p}_{15}{x}_5\end{array} $$

where the state variables denote the concentrations of the components, with *x*_1_ as unbound surface IR, *x*_2_ as bound surface IR, *x*_3_ as bound-phosphorylated surface IR, *x*_4_ as bound-phosphorylated internalized IR, and *x*_5_ as unbound internalized IR. The control variable *u* is considered as a constant insulin input (100 nM), while the initial condition of Eq. (46) is given as *x*_1_(0) = 100 %, and *x*_*i*_(0) = 0 for *i* ≠ 1 [49].

Since the control variable *u* is a constant, the nonlinear term *p*_21_*u* in Eq. (46) can be regarded as a parameter *p*_21_^′^ to be estimated. As a result, Eq. (46) becomes a linear model which can be analyzed by our method. Here, we consider three measured datasets used in [49] for parameter estimation, i.e. IR autophosphorylation from [61], IR internalization from [55], and remaining surface IR from [55]. These measured species are mixtures of the components denoted as state variables in Eq. (46), respectively, leading to the following output equations [49]

47 $$ {y}_1={x}_3+{x}_4 $$

48 $$ {y}_2={x}_4+{x}_5 $$

49 $$ {y}_3={x}_2+{x}_3 $$

where *y*_1_ is the percentage of total (surface and intracellular) phosphorylated IR, *y*_2_ is the percentage of total internalized IR, *y*_3_ is the percentage of total IR on the cell surface.

We are concerned with the identifiability of the 7 parameters in Eq. (46), when one or a combination of the above output equations is used for parameter estimation. Based on our method, it is found that, when only Eq. (47) is employed as an output equation, 4 parameters (i.e. *p*_21_^′^, *p*_51_, *p*_12_, *p*_32_) are non-identifiable, while 3 parameters (i.e. *p*_43_, *p*_54_, *p*_15_) are identifiable. In particular, the relationship of the 4 correlated parameters is expressed as follows

50 $$ \begin{array}{l}\frac{\partial {Y}_1(s)}{\partial {p}_{21}^{\prime }}+\frac{p_{51}}{\left({p}_{12}-{p}_{15}+{p}_{32}-{p}_{51}\right)}\frac{\partial {Y}_1(s)}{\partial {p}_{51}}\\ {}-\frac{\left({p}_{12}{p}_{21}^{\prime }-{p}_{12}{p}_{32}-{p}_{15}{p}_{21}^{\prime }+{p}_{15}{p}_{32}+{p}_{21}^{\prime }{p}_{32}-{p}_{32}^2+{p}_{32}{p}_{51}\right)}{p_{21}^{\prime}\left({p}_{12}-{p}_{15}+{p}_{32}-{p}_{51}\right)}\frac{\partial {Y}_1(s)}{\partial {p}_{12}}-\frac{p_{32}}{p_{21}^{\prime }}\frac{\partial {Y}_1(s)}{\partial {p}_{32}}=0\end{array} $$

This means that, using a dataset of IR autophosphorylation (i.e. *y*_1_ = *x*_3_ + *x*_4_), it is impossible to estimate all of the parameters of the model (even if the data are noise-free). Nevertheless, our computation results show that all of the 7 parameters are identifiable, i.e. there is no parameter correlation, when either Eq. (48) or Eq. (49) is used as an output equation. As a result, either a dataset of the IR internalization (i.e. *y*_2_ = *x*_4_ + *x*_5_) or a dataset of the remaining surface IR (i.e. *y*_3_ = *x*_2_ + *x*_3_) is enough for unique estimation of the 7 parameters of the model, when the measured data are noise-free. Obviously, unique estimation can also be achieved when a combination of the 3 output equations are used (i.e., simultaneously fitting the model to two or three datasets from [55] and [61]), as was performed in [49].

## Conclusions

A partial observation of state variables usually leads to non-identifiable parameters even for pretty simple models. To address this problem, a method for identifying parameter correlations in partially observed linear dynamic models is presented in this paper. The basic idea is to derive the output sensitivity matrix and analyze the linear dependences of the columns in this matrix. Thus the method is quite simple, i.e. only the Laplace transformation and linear algebra are required to derive the results. A special feature of our method is its explicit coupling of parameter correlations to control signals and the initial condition which can be used for experimental design, so that proper (noise-free) datasets can be generated for unique parameter estimation. In this way, the practically non-identifiable parameters can be estimated. Several partially observed linear compartmental models are used to demonstrate the capability of the proposed method for identifying the parameter correlations. Results derived from our method are verified by numerical parameter estimation. The extension of this method to partially observed nonlinear models could be a future study.

## Additional file

Additional file 1:
**Derivation of the sensitivity matrix and solutions of the homogeneous linear equations in example 1, 2 and 4.** This file contains detailed derivations and descriptions of the methods and associated results of the examples. (PDF 190 kb)

## References

[1] O’Brien EJ, Palsson BO (2015). Computing the functional proteome: recent progress and future prospects for genome-scale models. Curr Opin Biotech.

[2] Gunawardena J (2014). Models in biology: ‘accurate descriptions of our pathetic thinking’. BMC Biol.

[3] Bartl M, Kötzing M, Schuster S, Li P, Kaleta C (2013). Dynamic optimization identifies optimal programmes for pathway regulation in prokaryotes. Nat Commun.

[4] Wessely F, Bartl M, Guthke R, Li P, Schuster S, Kaleta C. Optimal regulatory strategies for metabolic pathways in Escherichia coli depending on protein costs. Mol Sys Biol. 2011;7:515. 10.1038/msb.2011.46 PMC3159982 21772263

[5] Eydgahi H, Chen WW, Muhlich JL, Vitkup D, Tsitsiklis JN, Sorger PK (2013). Properties of cell death models calibrated and compared using Bayesian approaches. Mol Sys Biol.

[6] Tummler K, Lubitz T, Schelker M, Klipp E (2013). New types of experimental data shape the use of enzyme kinetics for dynamic network modeling. FEBS J.

[7] Kreutz C, Timmer J (2009). Systems biology: experimental design. FEBS J.

[8] Bachmann J, Raue A, Schilling M, Böhm ME, Kreutz C, Kaschek D, Busch H, Gretz N, Lehmann WD, Timmer J, Klingmüller U (2011). Division of labor by dual feedback regulators controls JAK2/STAT5 signaling over broad ligand range. Mol Sys Biol.

[9] Chu Y, Hahn J (2009). Parameter selection via clustering of parameters into pairwise indistinguishable groups of parameters. Ind Eng Chem Res.

[10] Cracium G, Pantea C (2008). Identifiability of chemical reaction networks. J Math Chem.

[11] Wiechert W, Noack S (2011). Mechanistic pathway modeling for industrial biotechnology: challenging but worthwhile. Curr Opin Biotech.

[12] Heijnen JJ, Verheijen PJT (2013). Parameter identification of in vivo kinetic models: Limitations and challenges. Biotechnol J.

[13] Link H, Christodoulou D, Sauer U (2013). Advancing metabolic models with kinetic information. Curr Opin Biotech.

[14] McLean KAP, McAuley KB (2012). Mathematical modelling of chemical processes-obtaining the best model predictions and parameter estimates using identifiability and estimability procedures. Can J Chem Eng.

[15] Li P, Vu QD (2013). Identification of parameter correlations for parameter estimation in dynamic biological models. BMC Syst Biol.

[16] Raue A, Kreutz C, Maiwald T, Bachmann J, Schilling M, Klingmüller U, Timmer J (2009). Structural and practical identifiability analysis of partially observable dynamical models by exploiting the profile likelihood. Bioinformatics.

[17] Steiert B, Raue A, Timmer J, Kreutz C (2012). Experimental design for parameter estimation of gene regulatory networks. PLoS ONE.

[18] Chen WW, Schoeberl B, Jasper PJ, Niepel M, Nielsen UB, Lauffenburger DA, Sorger PK (2009). Input–output behavior of ErbB signaling pathways as revealed by a mass action model trained against dynamic data. Mol Sys Biol.

[19] Achard P, De Schutter E (2006). Complex parameter landscape for a complex neuron model. PloS Comput Biol.

[20] Gutenkunst RN, Waterfall JJ, Casey FP, Brown KS, Myers CR, Sethna JP (2007). Universally sloppy parameter sensitivities in systems biology models. PloS Comp Biol.

[21] Ashyraliyev M, Fomekong-Nanfack Y, Kaandorp JA, Blom JG (2009). Systems biology: parameter estimation for biochemical models. FEBS J.

[22] Chou IC, Voit EO (2009). Recent developments in parameter estimation and structure identification of biochemical and genomic systems. Math Biosci.

[23] Villaverde AF, Banga JR (2014). Reverse engineering and identification in systems biology: strategies, perspectives and challenges. J R Soc Interface.

[24] Lamberton TO, Condon ND, Stow JL, Hamilton NA (2014). On linear models and parameter identifiability in experimental biological systems. J Theor Biol.

[25] Bellman R, Aström KJ (1970). On structural identifiability. Math Biosci.

[26] Audoly S, D’Angio L (1983). On the identifiability of linear compartmental systems: a revisited transfer function approach based on topological properties. Math Biosci.

[27] Godfrey KR, Chapman ML (1990). Identifiability and indistinguishability of linear compartmental models. Math Comp Simul.

[28] Ljung L, Glad T (1994). On global identifiability for arbitrary model parametrizations. Automatica.

[29] Audoly S, D’Angio L, Saccomani MP, Cobelli C (1998). Global identifiability of linear compartmental models – a computer algebra algorithm. IEEE Trans Biomed Eng.

[30] Godfrey KR, Jones RP, Brown RF, Norton JP (1982). Factors Affecting the Identifiability of Compartmental Models. Automatica.

[31] Kachanov BO (2009). Symmetric Laplace transform and its application to parametric identification of linear systems. Automat Rem Contr.

[32] Meshkat N, Sullivant S (2014). Identifiable reparametrizations of linear compartment models. J Symbol Comp.

[33] Evans ND, Chappell MJ (2000). Extensions to a procedure for generating locally identifiable reparameterisations of unidentifiable systems. Math Biosci.

[34] Eisenberg MC, Hayashi MAL (2014). Determining identifiable parameter combinations using subset profiling. Math Biosci.

[35] Meshkat N, Kuo CE, DiStefano J (2014). On finding and using identifiable parameter combinations in nonlinear dynamic systems biology models and COMBOS: a novel web implementation. PloS ONE.

[36] Stanhope S, Rubin JE, Swigon D (2014). Identifiabiliy of linear and linear-in-parameters dynamical systems from a single trajectory. SIAM J App Dyn Syst.

[37] Chappel MJ, Godfrey KR, Vajda S (1990). Global identifiability of the parameters of nonlinear systems with specified inputs: a comparison of methods. Math Biosci.

[38] Chis OT, Banga JR, Balsa-Canto E (2011). Structural identifiability of systems biology models: a critical comparison of methods. PloS ONE.

[39] Meshkat N, Eisenberg M, DiStefano J (2009). An algorithm for finding globally identifiable parameter combinations of nonlinear ODE models using Gröbner bases. Math Biosci.

[40] Cole DJ, Morgan BJT, Titterington DM (2010). Determining the parametric structure of models. Math Biosci.

[41] Chis O, Banga JR, Balsa-Canto E (2011). GenSSI: a software toolbox for structural identifiability analysis of biological models. Bioinformatics.

[42] Bellu G, Saccomani MP, Audoly S, D’Angio L (2007). DAISY: A new software tool to test global identifiability of biological and physiological systems. Comp Meth Prog Biomed.

[43] Saccomani MP, Audoly S, D’Angio L (2003). Parameter identifiability of nonlinear systems: the role of initial conditions. Automatica.

[44] Hengl S, Kreutz C, Timmer J, Maiwald T (2007). Data-based identifiability analysis of nonlinear dynamical models. Bioinformatics.

[45] Roper R, Saccomani MP, Vicini P (2010). Cellular signaling identifiabilities: a case study. J Theor Biol.

[46] Faber R, Li P, Wozny G (2003). Sequential parameter estimation for large-scale systems with multiple data sets. I: computational framework. Ind Eng Chem Res.

[47] Zhao C, Vu QD, Li P (2013). A quasi-sequential parameter estimation for nonlinear dynamic systems based on multiple data profiles. Korean J Chem Eng.

[48] Vu QD. Parameter estimation in complex nonlinear dynamical systems. PhD Thesis, Technische Universität Ilmenau, 2015.

[49] Hori S, Kurland IJ, DiStenfano JJ (2006). Role of endosomal trafficking dynamics on the regulation of hepatic insulin receptor activity: model for Fao cells. Ann Biomed Eng.

[50] White MF (1997). The insulin signaling system and IRS proteins. Diabetologia.

[51] Taha C, Klip A (1999). The insulin signaling pathway. J Membrane Biol.

[52] Knutson VP (1991). Cellular trafficking and processing of the insulin receptor. FASEB J.

[53] Corin R, Donner D (1982). Insulin receptors convert to a higher affinity state subsequent to hormone binding. A two-state model for the insulin receptor. J Biol Chem.

[54] Standaert MJ, Pollet RJ (1984). Equilibrium model for insulin-induced receptor down-regulation. Regulation of insulin receptors in differentiated BC3H-I myocytes. J Biol Chem.

[55] Backer J, Kahn C, White MF (1989). Tyrosine phosphorylation of the insulin receptor during insulin-stimulated internalization in rat hepatoma cells. J Biol Chem.

[56] Quon MJ, Campfield L (1991). A mathematical model and computer simulation study of insulin receptor regulation. J Theor Biol.

[57] Wanant S, Quon MJ (2000). Insulin receptor binding kinetics: modeling and simulation studies. J Theor Biol.

[58] Sedaghat AR, Sherman A, Quon MJ (2002). A mathematical model of metabolic insulin signaling pathways. Am J Physiol Endocrinol Metab.

[59] Koschorreck M, Gilles ED (2008). Mathematical modeling and analysis of insulin clearance in vivo. BMC Syst Biol.

[60] Ho CK, Rahib L, Liao JC, Sriram G, Dipple KM (2015). Mathematical modeling of the insulin signal transduction pathway for prediction of insulin sensitivity from expression data. Mol Gen Metab.

[61] White MF, Haring HU, Kasuga M, Kahn CR (1984). Kinetic properties and sites of autophosphorylation of partially purified insulin receptor from hepatoma cells. J Biol Chem.

[62] Backer JM, Kahn CR, White MF (1989). Tyrosine phosphorylation of the insulin receptor is not required for receptor internalization: studies in 2,4-dinitrophenol-treated cells. Proc Natl Acad Sci USA.

