# Supplementary material for: A simple method for identifying parameter correlations in partially observed linear dynamic models
Source: BMC Syst Biol. 2015 Dec 14;9:92. doi: 10.1186/s12918-015-0234-3 (PMC4678707; doi:10.1186/s12918-015-0234-3)
Supplement: Additional file 1: — Derivation of the sensitivity matrix and solutions of the homogeneous linear equations in example 1, 2 and 4. This file contains detailed derivations and descriptions of the methods and associated results of the examples. (PDF 190 kb) [file 12918_2015_234_MOESM1_ESM.pdf]

Additional file 1 for

A simple method for identifying parameter correlations in partially observed linear models

Pu Li and Quoc Dong Vu

Derivation of the sensitivity matrix and solutions of the homogeneous linear equations in example 1, 2 and 4

1) Derivation of the output sensitivity matrix

The output sensitivities to the parameters in the individual matrices are as follows

$$\frac{\partial \mathbf{Y}(s)}{\partial \mathbf{p}_A} = \mathbf{C}\left(s\mathbf{I} - \mathbf{A}\right)^{-1} \mathbf{M}_A(\mathbf{X}(s)) \tag{A1}$$

$$\frac{\partial \mathbf{Y}(s)}{\partial \mathbf{p}_B} = \mathbf{C}\left(s\mathbf{I} - \mathbf{A}\right)^{-1} \mathbf{M}_B(\mathbf{U}(s)) \tag{A2}$$

$$\frac{\partial \mathbf{Y}(s)}{\partial \mathbf{p}_C} = \mathbf{M}_C(\mathbf{X}(s)) \tag{A3}$$

$$\frac{\partial \mathbf{Y}(s)}{\partial \mathbf{p}_D} = \mathbf{M}_D(\mathbf{U}(s)) \tag{A4}$$

where the state variables can be expressed as

$$\mathbf{X}(s) = \frac{1}{\det(s\mathbf{I} - \mathbf{A})}\left(adj\left(s\mathbf{I} - \mathbf{A}\right)\right)\left(\mathbf{B}\mathbf{U}(s) + \mathbf{x}_0\right) \tag{A5}$$

From Eq. (A1)

$$\begin{aligned} \frac{\partial \mathbf{Y}(s)}{\partial \mathbf{p}_A} &= \frac{1}{\det(s\mathbf{I} - \mathbf{A})}\left(\mathbf{C}\left(adj\left(s\mathbf{I} - \mathbf{A}\right)\right)\mathbf{M}_A(\mathbf{X}(s))\right) \\ &= \frac{1}{\left(\det(s\mathbf{I} - \mathbf{A})\right)^2}\left(\mathbf{C}\left(adj\left(s\mathbf{I} - \mathbf{A}\right)\right)\mathbf{M}_A\left(\left(adj\left(s\mathbf{I} - \mathbf{A}\right)\right)\left(\mathbf{B}\mathbf{U}(s) + \mathbf{x}_0\right)\right)\right) \end{aligned} \tag{A6}$$

From Eq. (A2)

$$\frac{\partial \mathbf{Y}(s)}{\partial \mathbf{p}_B} = \frac{1}{\det(s\mathbf{I} - \mathbf{A})}\left(\mathbf{C}\left(adj\left(s\mathbf{I} - \mathbf{A}\right)\right)\mathbf{M}_B(\mathbf{U}(s))\right)$$

Thus the output sensitivity matrix has the following form

$$\frac{\partial \mathbf{Y}(s)}{\partial \mathbf{p}} = \frac{1}{\left(\det(s\mathbf{I}-\mathbf{A})\right)^2} \begin{pmatrix} \mathbf{C}\left(adj\left(s\mathbf{I}-\mathbf{A}\right)\right)\mathbf{M}_A\left(\left(adj\left(s\mathbf{I}-\mathbf{A}\right)\right)\left(\mathbf{B}\mathbf{U}(s)+\mathbf{x}_0\right)\right) \\ \det(s\mathbf{I}-\mathbf{A})\mathbf{C}\left(adj\left(s\mathbf{I}-\mathbf{A}\right)\right)\mathbf{M}_B(\mathbf{U}(s)) \\ \det(s\mathbf{I}-\mathbf{A})\mathbf{M}_C(\left(adj\left(s\mathbf{I}-\mathbf{A}\right)\right)\left(\mathbf{B}\mathbf{U}(s)+\mathbf{x}_0\right)) \\ \left(\det(s\mathbf{I}-\mathbf{A})\right)^2\mathbf{M}_D(\mathbf{U}(s)) \end{pmatrix}^T \tag{A7}$$

Let

$$\begin{aligned} \mathbf{Q}_A(s) &= \mathbf{C}\left(adj\left(s\mathbf{I}-\mathbf{A}\right)\right)\mathbf{M}_A\left(\left(adj\left(s\mathbf{I}-\mathbf{A}\right)\right)\left(\mathbf{B}\mathbf{U}(s)+\mathbf{x}_0\right)\right) \\ \mathbf{Q}_B(s) &= \det(s\mathbf{I}-\mathbf{A})\mathbf{C}\left(adj\left(s\mathbf{I}-\mathbf{A}\right)\right)\mathbf{M}_B(\mathbf{U}(s)) \\ \mathbf{Q}_C(s) &= \det(s\mathbf{I}-\mathbf{A})\mathbf{M}_C\left(\left(adj\left(s\mathbf{I}-\mathbf{A}\right)\right)\left(\mathbf{B}\mathbf{U}(s)+\mathbf{x}_0\right)\right) \\ \mathbf{Q}_D(s) &= \left(\det(s\mathbf{I}-\mathbf{A})\right)^2\mathbf{M}_D(\mathbf{U}(s)) \end{aligned} \tag{A8}$$

In (A8), $\mathbf{A},\mathbf{B},\mathbf{C},\mathbf{D}$ are constant matrices, $\mathbf{x}_0$ is a constant vector, $\mathbf{U}(s)$ is the input vector. $\det(s\mathbf{I}-\mathbf{A})$ is a polynomial with the highest order of n_x. The elements in the adjunct matrix $adj\left(s\mathbf{I}-\mathbf{A}\right)$ are polynomials with the highest order of n_x-1. Therefore, the highest order of the polynomials of the elements in the matrices $\mathbf{Q}_A(s),\mathbf{Q}_B(s),\mathbf{Q}_C(s),\mathbf{Q}_D(s)$ will be $2(n_x-1)$, $2n_x-1$, $2n_x-1$, $2n_x$, respectively. The coefficients of the terms of these polynomials are functions of the parameters in the matrices $\mathbf{A},\mathbf{B},\mathbf{C},\mathbf{D}$, the elements of the input vector $\mathbf{U}(s)$ as well as the elements of the initial state vector $\mathbf{x}_0$.

2) Solutions of the linear homogeneous equations in Example 1

In this example, the polynomials in the output sensitivity vector are

$$\begin{aligned} q_1(s) &= V(s+p_3+p_4)\left((s+p_3+p_4)(U(s)+x_{10})+p_3x_{20}\right) \\ q_2(s) &= V(s+p_4)\left((s+p_3+p_4)(U(s)+x_{10})+p_3x_{20}\right) \\ q_3(s) &= -V(s+p_4)\left(p_2(U(s)+x_{10})+(s+p_1+p_2)x_{20}\right) \\ q_4(s) &= Vp_3\left(p_2(U(s)+x_{10})+(s+p_1+p_2)x_{20}\right) \\ q_5(s) &= \left((s+p_3+p_4)(U(s)+x_{10})+p_3x_{20}\right)\left((s+p_1+p_2)(s+p_3+p_4)-p_2p_3\right) \end{aligned} \tag{A9}$$

Now we define $\boldsymbol{\alpha}=(\alpha_1,\cdots,\alpha_5)^T$ and let

$$\alpha_1q_1(s)+\cdots+\alpha_5q_5(s)=0 \tag{A10}$$

It follows a polynomial equation with the highest order of 3 which leads to the following 4 homogenous linear equations with ($\alpha_1,\cdots,\alpha_5$) as unknowns

$$
\begin{aligned}
&\alpha_5 = 0 \\
&(U(s)+x_{10})(\alpha_1+\alpha_2) - x_{20}\alpha_3 = 0 \\
&\begin{pmatrix} \left(2(p_3+p_4)(U(s)+x_{10}) + p_3x_{20}\right)\alpha_1 + \left((p_3+2p_4)(U(s)+x_{10}) + p_3x_{20}\right)\alpha_2 \\ -(p_2(U(s)+x_{10}) + (p_1+p_2)x_{20})\alpha_3 + p_3x_{20}\alpha_4 \end{pmatrix} = 0 \\
&\begin{pmatrix} \left((p_3+p_4)^2(U(s)+x_{10}) + (p_3+p_4)p_3x_{20}\right)\alpha_1 + \left(p_4(p_3+p_4)(U(s)+x_{10}) + p_3p_4x_{20}\right)\alpha_2 \\ -\left(p_2p_4(U(s)+x_{10}) + p_4(p_1+p_2)x_{20}\right)\alpha_3 + \left(p_2p_3(U(s)+x_{10}) + (p_1+p_2)p_3x_{20}\right)\alpha_4 \end{pmatrix} = 0
\end{aligned} \tag{A11}
$$

Since the difference of the number of unknowns to the number of equations is one, there will be correlations among the parameters. From Eq. (A11), $\alpha_5 = 0$ means the parameter V is uncorrelated with the other parameters.

Considering the case of $U(s)=0$, the remaining equations in Eq. (A11) can be rewritten as follows

$$
\begin{aligned}
&\begin{pmatrix} x_{10} & x_{10} & -x_{20} \\ 2(k_3+k_4)x_{10} + k_3x_{20} & (k_3+2k_4)x_{10} + k_3x_{20} & -(k_2x_{10} + (k_1+k_2+k_4)x_{20}) \\ (k_3+k_4)^2x_{10} + k_3(k_3+k_4)x_{20} & k_4(k_3+k_4)x_{10} + k_3k_4x_{20} & -(k_2k_4x_{10} + (k_1+k_2)k_4x_{20}) \end{pmatrix} \begin{pmatrix} \alpha_1 \\ \alpha_2 \\ \alpha_3 \end{pmatrix} \\
&= \begin{pmatrix} 0 \\ -k_3x_{20} \\ -\left(k_3k_2x_{10} + (k_1+k_2)k_3x_{20}\right) \end{pmatrix} \alpha_4
\end{aligned} \tag{A12}
$$

If $x_{10} \neq 0$ and $x_{20} = 0$, then Eq. (A12) reduces to

$$
\begin{aligned}
&\alpha_1 + \alpha_2 = 0 \\
&2(p_3+p_4)\alpha_1 + (p_3+2p_4)\alpha_2 - p_2\alpha_3 = 0 \\
&(p_3+p_4)^2\alpha_1 + k_4(p_3+p_4)\alpha_2 - p_2p_4\alpha_3 + p_2p_3\alpha_4 = 0
\end{aligned} \tag{A13}
$$

Solving Eq. (A13) we have

$$
\alpha_1 = -\frac{p_2}{p_3}\alpha_4, \quad \alpha_2 = \frac{p_2}{p_3}\alpha_4, \quad \alpha_3 = -\alpha_4 \tag{A14}
$$

If $x_{10} = 0$ and $x_{20} \neq 0$, then Eq. (A12) reduces to

$$
\begin{aligned}
&\alpha_3 = 0 \\
&\alpha_1 + \alpha_2 + \alpha_4 = 0 \\
&(p_3+p_4)\alpha_1 + p_4\alpha_2 + (p_1+p_2)\alpha_4 = 0
\end{aligned} \tag{A15}
$$

The solution of Eq. (A15) leads to

$$
\alpha_1 = \frac{1}{p_3}(p_4 - p_1 - p_2)\alpha_4, \quad \alpha_2 = \frac{1}{p_3}(p_1 + p_2 - p_3 - p_4)\alpha_4, \quad \alpha_3 = 0 \tag{A16}
$$

If $x_{10} \neq 0$ and $x_{20} \neq 0$, then the solution of the 3 linear equations in Eq. (A12) leads to $\alpha_1, \alpha_2, \alpha_3$ with respect to α_4. However, the resulting relations depend on $x_{10} \neq 0$ and $x_{20} \neq 0$.

3) Solutions of the linear homogeneous equations in Example 2

In this example we have

$$\mathbf{A}=\begin{pmatrix}-p_{21} & p_{12} & p_{13}\\ p_{21} & -p_{12} & 0\\ 0 & 0 & -p_{13}\end{pmatrix},\quad \mathbf{B}=\begin{pmatrix}1\\0\\0\end{pmatrix},\quad \mathbf{C}=\begin{pmatrix}0 & 1 & 0\end{pmatrix} \tag{A14}$$

and

$$\mathbf{M}_A(\mathbf{X}(s))=\begin{pmatrix}-X_1(s) & X_2(s) & X_3(s)\\ X_1(s) & -X_2(s) & 0\\ 0 & 0 & -X_3(s)\end{pmatrix} \tag{A15}$$

Since

$$(s\mathbf{I}-\mathbf{A})^{-1}=\frac{1}{\Delta}\begin{pmatrix}(s+p_{12})(s+p_{13}) & p_{12}(s+p_{13}) & p_{13}(s+p_{12})\\ k_{21}(s+p_{13}) & (s+p_{21})(s+p_{13}) & p_{13}p_{21}\\ 0 & 0 & (s+p_{21})(s+p_{12})-p_{12}p_{21}\end{pmatrix} \tag{A16}$$

where $\Delta=(s+p_{21})(s+p_{12})(s+p_{13})-p_{12}p_{21}(s+p_{13})=s(s+p_{13})(s+p_{12}+p_{21})$, then the output sensitivity vector

$$\begin{aligned}\frac{\partial Y(s)}{\partial \mathbf{p}}&=\mathbf{C}\left(s\mathbf{I}-\mathbf{A}\right)^{-1}\mathbf{M}_A(\mathbf{X}(s))\\
&=\begin{pmatrix}0 & 1 & 0\end{pmatrix}\frac{1}{\Delta}\begin{pmatrix}(s+p_{12})(s+p_{13}) & p_{12}(s+p_{13}) & p_{13}(s+p_{12})\\ p_{21}(s+p_{13}) & (s+p_{21})(s+p_{13}) & p_{13}p_{21}\\ 0 & 0 & (s+p_{21})(s+p_{12})-p_{12}p_{21}\end{pmatrix}\mathbf{M}_A(\mathbf{X}(s))\\
&=\frac{1}{\Delta}\begin{pmatrix}p_{21}(s+p_{13}) & (s+p_{21})(s+p_{13}) & p_{13}p_{21}\end{pmatrix}\begin{pmatrix}-X_1(s) & X_2(s) & X_3(s)\\ X_1(s) & -X_2(s) & 0\\ 0 & 0 & -X_3(s)\end{pmatrix}\\
&=\frac{s}{\Delta}\begin{pmatrix}(s+p_{13})X_1(s) & -(s+p_{13})X_2(s) & p_{21}X_3(s)\end{pmatrix}\end{aligned} \tag{A17}$$

The solution of the state equations leads to

$$\begin{aligned}\begin{pmatrix}X_1(s)\\ X_2(s)\\ X_3(s)\end{pmatrix}&=(sI-A)^{-1}\begin{pmatrix}U(s)+x_{10}\\ x_{20}\\ x_{30}\end{pmatrix}\\
&=\frac{1}{\Delta}\begin{pmatrix}\left(U(s)+x_{10}\right)s^2+\left((p_{12}+p_{13})\left(U(s)+x_{10}\right)+p_{12}x_{20}+p_{13}x_{30}\right)s+p_{12}p_{13}\left(U(s)+x_{10}+x_{20}+x_{30}\right)\\ x_{20}s^2+\left((p_{21}+p_{13})x_{20}+p_{21}\left(U(s)+x_{10}\right)\right)s+p_{13}p_{21}\left(U(s)+x_{10}+x_{20}+x_{30}\right)\\ s(s+p_{12}+p_{21})x_{30}\end{pmatrix}\end{aligned} \tag{A18}$$

Then

$$\frac{\partial Y(s)}{\partial \mathbf{p}} = \frac{s}{\Delta}\begin{pmatrix}(s+p_{13})X_1(s) & -(s+p_{13})X_2(s) & p_{21}X_3(s)\end{pmatrix}$$

$$= \frac{s}{\Delta^2}\begin{pmatrix} (s+p_{13})\left(\left(U(s)+x_{10}\right)s^2 + \left((p_{12}+p_{13})\left(U(s)+x_{10}\right)+p_{12}x_{20}+p_{13}x_{30}\right)s + p_{12}p_{13}\left(U(s)+x_{10}+x_{20}+x_{30}\right)\right) \\ -(s+p_{13})\left(x_{20}s^2 + \left((p_{21}+p_{13})x_{20}+p_{21}\left(U(s)+x_{10}\right)\right)s + p_{13}p_{21}\left(U(s)+x_{10}+x_{20}+x_{30}\right)\right) \\ p_{21}x_{30}(s+p_{12}+p_{21})s \end{pmatrix}^T$$

(A19)

The elements in the output sensitivity vector

$$\begin{aligned} q_1(s) &= (s+p_{13})\left(\left(U(s)+x_{10}\right)s^2 + \left((p_{12}+k_{13})\left(U(s)+x_{10}\right)+p_{12}x_{20}+p_{13}x_{30}\right)s + p_{12}p_{13}\left(U(s)+x_{10}+x_{20}+x_{30}\right)\right) \\ q_2(s) &= -(s+p_{13})\left(x_{20}s^2 + \left((p_{21}+p_{13})x_{20}+p_{21}\left(U(s)+x_{10}\right)\right)s + p_{13}p_{21}\left(U(s)+x_{10}+x_{20}+x_{30}\right)\right) \\ q_3(s) &= p_{21}x_{30}(s+p_{12}+p_{21})s \end{aligned}$$

(A20)

To check the linear dependencies, we introduce 3 constants $\alpha_1, \alpha_2, \alpha_3$ and let $\alpha_1 q_1 + \alpha_2 q_2 + q_3\alpha_3 = 0$. It follows

$$\begin{aligned} &\alpha_1(s+p_{13})\left(\left(U(s)+x_{10}\right)s^2 + \left((p_{12}+p_{13})\left(U(s)+x_{10}\right)+p_{12}x_{20}+p_{13}x_{30}\right)s + p_{12}k_{13}\left(U(s)+x_{10}+x_{20}+x_{30}\right)\right) - \\ &\alpha_2(s+p_{13})\left(x_{20}s^2 + \left((p_{21}+p_{13})x_{20}+p_{21}\left(U(s)+x_{10}\right)\right)s + p_{13}p_{21}\left(U(s)+x_{10}+x_{20}+x_{30}\right)\right) + \\ &\alpha_3 p_{21}x_{30}(s+p_{12}+p_{21})s = 0 \end{aligned}$$

(A21)

which can be expressed as

$$\begin{aligned} &\left(\left(U(s)+x_{10}\right)\alpha_1 - x_{20}\alpha_2\right)s^3 \\ &+\begin{pmatrix} \left((p_{12}+2p_{13})\left(U(s)+x_{10}\right)+p_{12}x_{20}+p_{13}x_{30}\right)\alpha_1 - \\ \left((p_{21}+2p_{13})x_{20}+p_{21}\left(U(s)+x_{10}\right)\right)\alpha_2 \\ +p_{21}x_{30}\alpha_3 \end{pmatrix}s^2 \\ &+\begin{pmatrix} \left(p_{13}\left((p_{12}+p_{13})\left(U(s)+x_{10}\right)+p_{12}x_{20}+p_{13}x_{30}\right)+p_{13}p_{12}\left(U(s)+x_{10}+x_{20}+x_{30}\right)\right)\alpha_1 \\ -\left(p_{13}\left((p_{21}+p_{13})x_{20}+p_{21}\left(U(s)+x_{10}\right)\right)+p_{13}p_{21}\left(U(s)+x_{10}+x_{20}+x_{30}\right)\right)\alpha_2 \\ +p_{21}(p_{12}+p_{21})x_{30}\alpha_3 \end{pmatrix}s \\ &+k_{13}^2\left(k_{12}\alpha_1 - k_{21}\alpha_2\right)\left(U(s)+x_{10}+x_{20}+x_{30}\right) = 0 \end{aligned}$$

(A22)

It follows

$$
\begin{aligned}
&\left(U(s)+x_{10}\right)\alpha_1 - x_{20}\alpha_2 = 0\\
&\left((k_{12}+2p_{13})\left(U(s)+x_{10}\right)+p_{12}x_{20}+p_{13}x_{30}\right)\alpha_1 - \left((p_{21}+2p_{13})x_{20}+p_{21}\left(U(s)+x_{10}\right)\right)\alpha_2 + p_{21}x_{30}\alpha_3 = 0\\
&\begin{pmatrix}
\left(p_{13}\left((p_{12}+p_{13})\left(U(s)+x_{10}\right)+p_{12}x_{20}+p_{13}x_{30}\right)+p_{13}p_{12}\left(U(s)+x_{10}+x_{20}+x_{30}\right)\right)\alpha_1\\
-\left(p_{13}\left((p_{21}+p_{13})x_{20}+p_{21}\left(U(s)+x_{10}\right)\right)+p_{13}p_{21}\left(U(s)+x_{10}+x_{20}+x_{30}\right)\right)\alpha_2\\
+p_{21}(p_{12}+p_{21})x_{30}\alpha_3
\end{pmatrix} = 0\\
&p_{12}\alpha_1 - p_{21}\alpha_2 = 0
\end{aligned}
$$

(A23)

4) Solutions of the linear homogeneous equations in Example 4

From the state equations of the model we have

$$
(s\mathbf{I}-\mathbf{A})^{-1} = \begin{pmatrix}
s+p_{31} & 0 & -p_{13} & 0\\
0 & s+p_{42} & 0 & -p_{24}\\
-p_{31} & 0 & s+(p_{03}+p_{13}+p_{43}) & 0\\
0 & -p_{42} & -p_{43} & s+(p_{04}+p_{24})
\end{pmatrix}^{-1} = \frac{1}{\Delta}\begin{pmatrix}
b_{11} & b_{12} & b_{13} & b_{14}\\
b_{21} & b_{22} & b_{23} & b_{24}\\
b_{31} & b_{31} & b_{33} & b_{34}\\
b_{41} & b_{42} & b_{43} & b_{44}
\end{pmatrix}
$$

(A24)

where $\Delta = \det(s\mathbf{I}-\mathbf{A})$ and

$$
\begin{aligned}
b_{11} &= \left(s+p_{42}\right)(s+p_{03}+p_{13}+p_{43})(s+p_{04}+p_{24}) - p_{24}p_{42}(s+p_{03}+p_{13}+p_{43})\\
b_{12} &= 0\\
b_{13} &= -p_{13}p_{24}p_{42} + p_{13}(s+p_{03}+p_{13}+p_{43})(s+p_{04}+p_{24})\\
b_{14} &= 0\\
b_{21} &= p_{24}p_{31}p_{43}\\
b_{22} &= (s+p_{31})(s+p_{03}+p_{13}+p_{43})(s+p_{04}+p_{24}) - p_{13}p_{31}(s+p_{04}+p_{24})\\
b_{23} &= p_{24}p_{43}(s+p_{31})\\
b_{24} &= -p_{13}p_{24}p_{31} + p_{24}(s+p_{31})(s+p_{03}+p_{13}+p_{43})\\
b_{31} &= -p_{24}p_{31}p_{42} + p_{31}(s+p_{42})(s+p_{04}+p_{24})\\
b_{32} &= 0\\
b_{33} &= (s+p_{31})(s+p_{42})(s+p_{04}+p_{24}) - p_{24}p_{42}(s+p_{31})\\
b_{34} &= 0\\
b_{41} &= p_{31}p_{43}(s+p_{42})\\
b_{42} &= -p_{13}p_{31}p_{42} + p_{42}(s+p_{31})(s+p_{03}+p_{13}+p_{43})\\
b_{43} &= p_{43}(s+p_{31})(s+p_{42})\\
b_{44} &= (s+p_{31})(s+p_{42})(s+p_{03}+p_{13}+p_{43}) - p_{13}p_{31}(s+p_{42})
\end{aligned}
$$

(A25)

The solution of the state equations with $x_{10} = x_{20} = x_{30} = 0$ leads to

$$\mathbf{X}(s)=\left(s\mathbf{I}-\mathbf{A}\right)^{-1}\left(\mathbf{BU}(s)+\mathbf{x}_0\right)=\frac{1}{\Delta}\begin{pmatrix} b_{11} & 0 & b_{13} & 0 \\ b_{21} & b_{22} & b_{23} & b_{24} \\ b_{31} & 0 & b_{33} & 0 \\ b_{41} & b_{42} & b_{43} & b_{44} \end{pmatrix}\begin{pmatrix} 1 \\ 0 \\ 0 \\ 0 \end{pmatrix}U(s)=\frac{1}{\Delta}\begin{pmatrix} b_{11} \\ b_{21} \\ b_{31} \\ b_{41} \end{pmatrix}U(s) \tag{A26}$$

Then the output sensitivity matrix

$$\frac{\partial \mathbf{Y}(s)}{\partial \mathbf{p}}=\frac{1}{\Delta}\begin{pmatrix} b_{11} & 0 & b_{13} & 0 \\ b_{21} & b_{22} & b_{23} & b_{24} \end{pmatrix}\begin{pmatrix} -X_1 & X_3 & 0 & 0 & 0 & 0 & 0 \\ 0 & 0 & -X_2 & X_4 & 0 & 0 & 0 \\ X_1 & -X_3 & 0 & 0 & -X_3 & -X_3 & 0 \\ 0 & 0 & x_2 & -X_4 & X_3 & 0 & -X_4 \end{pmatrix}$$

$$=\frac{1}{\Delta}\begin{pmatrix} -(b_{11}-b_{13})X_1 & (b_{11}-b_{13})X_3 & 0 & 0 & -b_{13}X_3 & -b_{13}X_3 & 0 \\ -(b_{21}-b_{23})X_1 & (b_{21}-b_{23})X_3 & -(b_{22}-b_{24})X_2 & (b_{22}-b_{24})X_4 & (b_{24}-b_{23})X_3 & -b_{23}X_3 & -b_{24}X_4 \end{pmatrix} \tag{A27}$$

Based on Eq. (A26) we have

$$\frac{\partial \mathbf{Y}(s)}{\partial \mathbf{p}}=\frac{1}{\Delta^2}\begin{pmatrix} -(b_{11}-b_{13})b_{11} & (b_{11}-b_{13})b_{31} & 0 & 0 & -b_{13}b_{31} & -b_{13}b_{31} & 0 \\ -(b_{21}-b_{23})b_{11} & (b_{21}-b_{23})b_{31} & -(b_{22}-b_{24})b_{21} & (b_{22}-b_{24})b_{41} & (b_{24}-b_{23})b_{31} & -b_{23}b_{31} & -b_{24}b_{41} \end{pmatrix} \tag{A28}$$

Now we introduce ($\alpha_1,\cdots,\alpha_7$) and let

$$\alpha_1\begin{pmatrix} \frac{\partial Y_1}{\partial k_{31}} \\ \frac{\partial Y_2}{\partial k_{31}} \end{pmatrix}+\alpha_2\begin{pmatrix} \frac{\partial Y_1}{\partial k_{13}} \\ \frac{\partial Y_2}{\partial k_{13}} \end{pmatrix}+\alpha_3\begin{pmatrix} \frac{\partial Y_1}{\partial k_{42}} \\ \frac{\partial Y_2}{\partial k_{42}} \end{pmatrix}+\alpha_4\begin{pmatrix} \frac{\partial Y_1}{\partial k_{24}} \\ \frac{\partial Y_2}{\partial k_{24}} \end{pmatrix}+\alpha_5\begin{pmatrix} \frac{\partial Y_1}{\partial k_{43}} \\ \frac{\partial Y_2}{\partial k_{43}} \end{pmatrix}+\alpha_6\begin{pmatrix} \frac{\partial Y_1}{\partial k_{03}} \\ \frac{\partial Y_2}{\partial k_{03}} \end{pmatrix}+\alpha_7\begin{pmatrix} \frac{\partial Y_1}{\partial k_{04}} \\ \frac{\partial Y_2}{\partial k_{04}} \end{pmatrix}=\begin{pmatrix} 0 \\ 0 \end{pmatrix} \tag{A29}$$

Since the first two columns of the matrix in Eq. (A28) are linearly independent, then $\alpha_1=\alpha_2=0$. From the 5th and 6th elements of the first row in Eq. (A28) we find $\alpha_5+\alpha_6=0$. Thus the second row of Eq. (A29) has the following form

$$-(b_{22}-b_{24})b_{21}\alpha_3+(b_{22}-b_{24})b_{41}\alpha_4+(b_{24}-b_{23})b_{31}\alpha_5-b_{23}b_{31}\alpha_6-b_{24}b_{41}\alpha_7=0 \tag{A30}$$

From the expressions of b_{22} and b_{24} in Eq. (A25), Eq. (A30) becomes

$$(s+p_{04})(b_{41}\alpha_4-b_{21}\alpha_3)+p_{24}(b_{31}\alpha_5-b_{41}\alpha_7)=0 \tag{A31}$$

Replace the expressions of b_{21},b_{31},b_{41} to the above equation, it follows

$$\begin{aligned}&(s+p_{04})(p_{31}p_{43}(s+p_{42})\alpha_4-p_{24}p_{31}p_{43}\alpha_3)+p_{31}p_{24}(s^2+(p_{42}+p_{04}+p_{24})s+p_{04}p_{42})\alpha_5\\&-p_{31}p_{43}p_{24}(s+p_{42})\alpha_7=0\end{aligned} \tag{A32}$$

which can be rewritten as

$$\begin{aligned}&(p_{43}\alpha_4 + p_{24}\alpha_5)s^2 + \\ &\big((p_{04}+p_{42})p_{43}\alpha_4 - p_{24}p_{43}\alpha_3 + p_{24}(p_{42}+p_{04}+p_{24})\alpha_5 - p_{24}p_{43}\alpha_7\big)s + \\ &p_{04}p_{42}p_{43}\alpha_4 - p_{24}p_{43}p_{04}\alpha_3 + p_{24}p_{04}p_{42}\alpha_5 - p_{24}p_{43}p_{42}\alpha_7 = 0\end{aligned} \quad \text{(A33)}$$

This means

$$\begin{aligned}&p_{43}\alpha_4 + p_{24}\alpha_5 = 0 \\ &(p_{04}+p_{42})p_{43}\alpha_4 - p_{24}p_{43}\alpha_3 + p_{24}(p_{42}+p_{04}+p_{24})\alpha_5 - p_{24}p_{43}\alpha_7 = 0 \\ &p_{04}p_{42}p_{43}\alpha_4 - p_{24}p_{43}p_{04}\alpha_3 + p_{24}p_{04}p_{42}\alpha_5 - p_{24}p_{43}p_{42}\alpha_7 = 0\end{aligned} \quad \text{(A34)}$$

Then we have

$$\alpha_3 = -\frac{p_{24}p_{42}}{p_{43}(p_{04}-p_{42})}\alpha_5, \quad \alpha_4 = -\frac{p_{24}}{p_{43}}\alpha_5, \quad \alpha_7 = \frac{p_{24}p_{04}}{p_{43}(p_{04}-p_{42})}\alpha_5 \quad \text{(A35)}$$

Now we derive the identifiable parameter combinations. Due to $\alpha_5 + \alpha_6 = 0$, we have

$$\frac{\partial Y_1}{\partial k_{43}} - \frac{\partial Y_1}{\partial k_{03}} = 0 \quad \text{(A36)}$$

which means $p_{03} + p_{43} = const.$ and then the second row of Eq. (A29) is expressed as

$$\alpha_3\frac{\partial Y_2}{\partial k_{42}} + \alpha_4\frac{\partial Y_2}{\partial k_{24}} + \alpha_7\frac{\partial Y_2}{\partial k_{04}} = 0 \quad \text{(A37)}$$

Due to Eq. (A35), Eq. (A37) can be reformulated as follows

$$p_{42}\left(\frac{\partial Y_2}{\partial k_{42}} - \frac{\partial Y_2}{\partial k_{24}}\right) - p_{04}\left(\frac{\partial Y_2}{\partial k_{04}} - \frac{\partial Y_2}{\partial k_{24}}\right) = 0 \quad \text{(A38)}$$

The solution of this equation leads to $p_{42}p_{04}(p_{42}+p_{04}+p_{24}) = const.$ In addition, from Eq. (A34) we have $\alpha_4 = -\frac{p_{24}}{p_{43}}\alpha_5$ which means

$$p_{24}\frac{\partial Y_2}{\partial k_{24}} - p_{43}\frac{\partial Y_2}{\partial k_{43}} = 0 \quad \text{(A39)}$$

And thus there will be $p_{24}p_{43} = const.$
